# Pollination Type Recognition from a Distance by the Ovary Is Revealed Through a Global Transcriptomic Analysis

**DOI:** 10.3390/plants8060185

**Published:** 2019-06-24

**Authors:** Valentin Joly, Faïza Tebbji, André Nantel, Daniel P. Matton

**Affiliations:** 1 Institut de Recherche en Biologie Végétale, Université de Montréal, Montréal, QC H1X 2B2, Canada; valentin.joly@umontreal.ca; 2 CRCHU de Québec, Université Laval, Québec, QC G1V 4G2, Canada; faiza.tebbji@crchudequebec.ulaval.ca; 3 National Research Council Canada, Montréal, QC H4P 2R2, Canada; andre.nantel@nrc-cnrc.gc.ca

**Keywords:** long distance signaling, pollen–pistil interactions, pollen-associated molecular signatures, postmating isolation barriers

## Abstract

Sexual reproduction in flowering plants involves intimate contact and continuous interactions between the growing pollen tube and the female reproductive structures. These interactions can trigger responses in distal regions of the flower well ahead of fertilization. While pollination-induced petal senescence has been studied extensively, less is known about how pollination is perceived at a distance in the ovary, and how specific this response is to various pollen genotypes. To address this question, we performed a global transcriptomic analysis in the ovary of a wild potato species, *Solanum chacoense*, at various time points following compatible, incompatible, and heterospecific pollinations. In all cases, pollen tube penetration in the stigma was initially perceived as a wounding aggression. Then, as the pollen tubes grew in the style, a growing number of genes became specific to each pollen genotype. Functional classification analyses revealed sharp differences in the response to compatible and heterospecific pollinations. For instance, the former induced reactive oxygen species (ROS)-related genes while the latter affected genes associated to ethylene signaling. In contrast, incompatible pollination remained more akin to a wound response. Our analysis reveals that every pollination type produces a specific molecular signature generating diversified and specific responses at a distance in the ovary in preparation for fertilization.

## 1. Introduction

In Angiosperms, sexual reproduction is initiated by pollen landing on the stigma papillae. After hydration, pollen grains produce a pollen tube (PT) that grows through the internal tissue of the carpel, guided by physical as well as chemotropic cues originating from both the style and the ovary to finally deliver its two sperm cells to the female gametophyte [1]. One sperm cell fuses with the egg cell forming the zygote while the second sperm cell fuses with the central cell to form the endosperm that surrounds and provides nutrients to the developing embryo. From the onset of pollen grains landing on a receptive stigma surface until effective fertilization, multiple interactions are initiated and a complex and intricate cross talk between the pollen and the pistil is established [2]. The decision to accept or reject the pollen starts with pollen capture and adhesion, followed by pollen hydration and germination. At this stage, pollen grains might already be rejected, as found in species expressing sporophytic self-compatibility (SI), like in the *Brassicaceae* family [3]. In species expressing gametophytic SI systems, like in the *Papaveraceae* and in the *Solanaceae*, PT recognition and rejection occurs either soon after pollen germination [4] or later on during PT growth in the transmitting tissue of the style [5], respectively. Being highly specialized structures, pollen [6,7,8,9,10], stigma/style [11,12,13,14], ovary [15,16,17,18,19] as well as individual cells within the ovule [20,21] express a specific transcriptome. During pollen–pistil interactions, continuous intimate contact and concomitant signal exchanges are bound to further modulate these transcriptomes. Since the first large-scale report of pistil-induced gene expression in the PT by Qin et al. in 2009 [22], several studies have investigated transcriptional changes taking place in pollinated pistils in the context of compatible [23,24,25], incompatible [26,27,28], and interspecific crosses [29,30,31,32,33].

In the abovementioned studies, the modulated genes were isolated from the tissues in direct contact, however there is still the question of what goes on in distal structures before PTs reach the ovary. Long-distance signaling during plant reproduction was described almost 150 years ago with the discovery of pollination-induced ovule maturation in orchid species [34,35,36], which was later found to be associated with interorgan ethylene signaling [37,38]. Pollination was also shown to be required to complete female gametophyte development in other species such as almond [39], maize [40], and tobacco [41]. Moreover, pollination is known to trigger several other physiological responses in the flower [42], including petal senescence in orchids [43] and *Petunia* [44], or changes in floral scent, for example in thistles [45]. Again, ethylene was identified or suggested as the mediator of this long-distance signaling [42].

Such responses in distal organs require the modulation of genes at a distance following pollination, well before PTs reach the ovules. Indeed, several studies revealed that pollination induces the expression of ethylene biosynthesis genes in the flowers of orchids [38,46], tomato [47], and tobacco [41]. Moreover, Lantin et al. [48] showed that the *SPP2* gene from the wild potato species *Solanum chacoense*, which encodes a dioxygenase, is activated at a distance in the ovary by both compatible pollination and stigma wounding. This first observation on a single gene prompted us to expand the analysis and explore the global transcriptional response of *S. chacoense* ovaries to pollination. Although comprehensive transcriptomic studies were performed recently on pollination-induced responses in corollas [49,50], no large-scale study has yet addressed the specific issue of long-distance communication between growing PTs and ovules.

In this work, we set out to understand how precisely the ovary can interpret pollination from a distance in preparation for fertilization, and how specific this response is to various pollination types. To address these questions, we have used a global transcriptomic approach to monitor gene expression profiles in *S. chacoense* ovaries at different times following conspecific compatible (CCP), conspecific incompatible (CIP), and heterospecific compatible (HCP) pollinations as well as from stigma wounding and touch treatments.

## 2. Results and Discussion

### 2.1. Experimental Design

We used an ovule cDNA microarray consisting of 7741 sequences representing 6374 unigenes [19] to globally analyze the ovule transcriptomic response following pollination. The microarray included cDNAs from various developmental stages, from unpollinated ovules (UOs) to fertilized ovules until late torpedo stage embryos, sequenced in the form of expressed sequence tags (ESTs) [51].

Since gametophytic SI is an important PT rejection mechanism in our model species, involving pollination-induced regulation of pistil transcripts [52,53,54], we chose to compare the effect of CCP and CIP on gene expression in the ovary. Moreover, the existence of cross-incompatibility barriers affecting pollen–pistil interactions in wild potatoes [55] led us to include a HCP condition in our study. To minimize incongruity problems, we chose to perform HCP with pollen from a closely related self-incompatible species, *S. microdontum*, which was previously shown to make fertile hybrids with *S. chacoense* [56]. Finally, to investigate the possible relationship between PT perception and wound or mechanical stress, we also included a stigma wounding (W) and a touch (T) condition in our design, the latter involving mock pollinations made with inert zirconia/silica microbeads.

**Figure 1:**
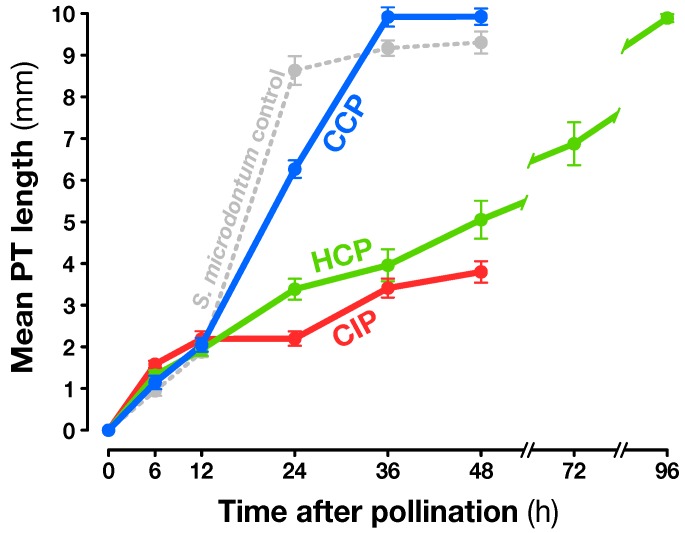
Pollen tube (PT) growth kinetics after conspecific compatible (CCP), conspecific incompatible (CIP), and heterospecific compatible (HCP) pollinations. PT length was measured after aniline blue staining of *S. chacoense* G4 pistils pollinated with *S. chacoense* G4 (CIP, red) and V22 (CCP, blue), as well as *S. microdontum* (HCP, green). The light gray line represents PT kinetics after intraspecific *S. microdontum* pollination.

To choose the best time points for the analysis, PT growth kinetics were monitored in vivo (Figure 1). The three PT types germinated equally and had undistinguishable growth until 12 h after pollination (HAP), where they all reached ∼2 mm in length. To determine if the ovary could accurately discriminate between pollination types before any visible difference in PT growth, 6 HAP was chosen as the first time point. Then, after growing slowly (∼170 $\mu m/h^{-1}$) until they emerged from the stigma 12 HAP, CCP PTs dramatically sped up (∼330 $\mu m/h^{-1}$) to finally exit the style around 30 HAP. This biphasic growth pattern is characteristic for species that shed bicellular pollen (containing a vegetative and a generative cell) like solanaceous species [57]. The first phase, termed the autotrophic phase, is characterized by a period of slow growth where PTs rely on their stored reserves. Next, the heterotrophic phase is characterized by a faster growth rate, the pollen being fed by nutrient made available from the stylar transmitting tissue. In *S. chacoense*, PTs normally reach the first available ovules a few hours later to effect fertilization [58].

Interestingly, CIP and HCP PTs displayed a steady but slower monophasic growth pattern. CIP PTs were all stopped by the SI reaction before they reached mid-style, whereas HCP PTs faced suboptimal growth in the heterospecific style, which is often described as incongruity [59]. A control pollination in *S. microdontum* (Figure 1, light gray line) confirmed that HCP PTs grow faster in their conspecific pistils. Since most CIP PTs were already arrested 24 HAP while CCP PTs had not yet reached the ovary, this was chosen as the second reference time point. Finally, in case interorgan signaling needed more time to be detected, a late time point, 48 HAP, was also selected to determine late pollination effects, especially for CIP and HCP. The same time points (6, 24, and 48 h) were used to examine transcript regulation after the wounding and touch treatments.

### 2.2. Expression Profiling of Pollination-Responsive Genes

For each time point in each pollination condition, four ovule samples were collected from a large number of plants grown in the same greenhouse. After RNA extraction and cDNA library construction, half of these biological replicates were labeled with Cy3 and the other half with Cy5 to account for the possibility of dye bias. Following the procedure used by Tebbji et al. [19], competitive hybridizations were made against the same pooled control obtained from different UO replicates. To confirm the reliability of this approach, six additional control hybridizations were made with individual UO replicates against the pooled control (Dataset S1a).

**Figure 2:**
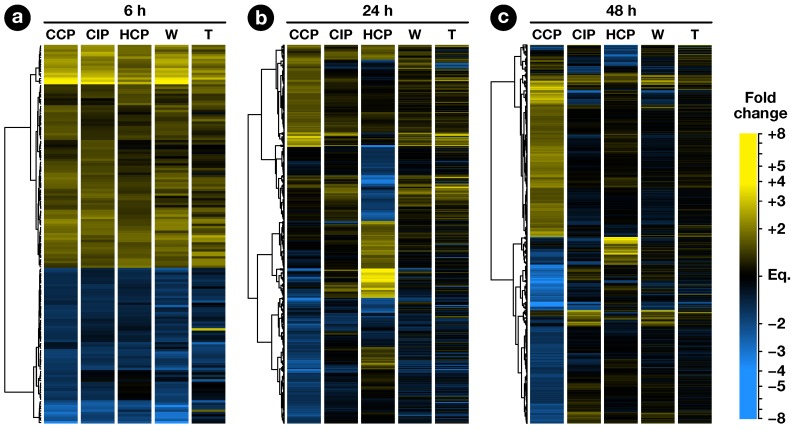
Hierarchical clustering analysis of regulated genes. Each row represents a gene and each column represents a condition. At each time point, the analysis was performed using genes regulated in at least one pollination condition. Euclidean distances between expression ratios in CCP, CIP and HCP vs. unpollinated ovule (UO) were used in a hierarchial clustering analysis based on Ward’s method. Expression ratios in stigma wounding (W) and touch (T) conditions vs. UO were then added to the heatmap.

**Figure 3:**
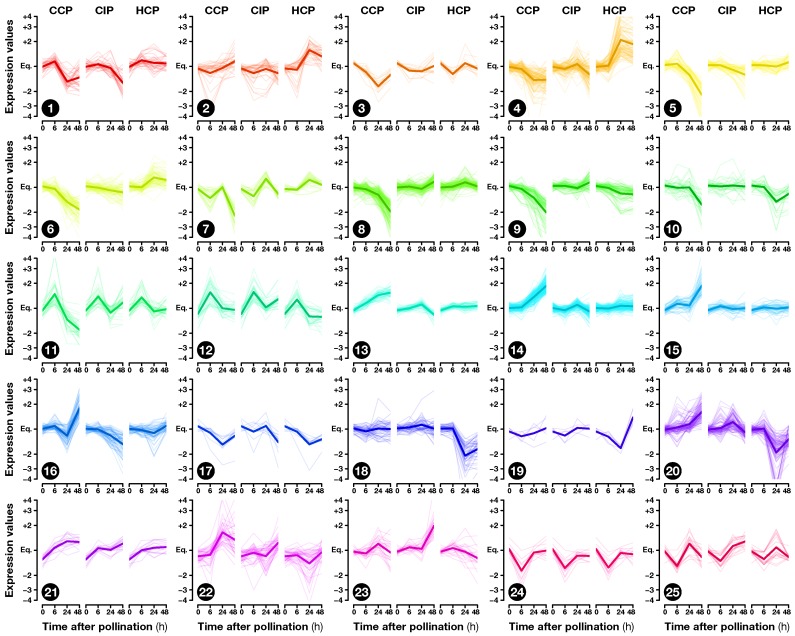
Transcription expression profiles in the 25 clusters obtained by *k*-means clustering.

**Figure 4:**
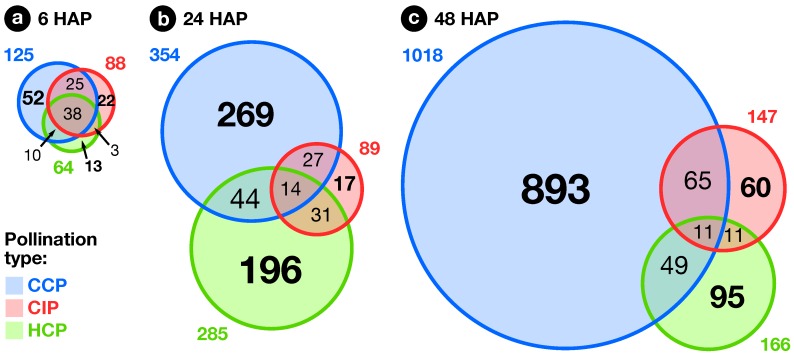
Overlap between pollination responses. Venn diagrams showing the overlap between CCP-, CIP-, and HCP-regulated genes at 6, 24 and 48 h after pollination (HAP).

**Figure 5:**
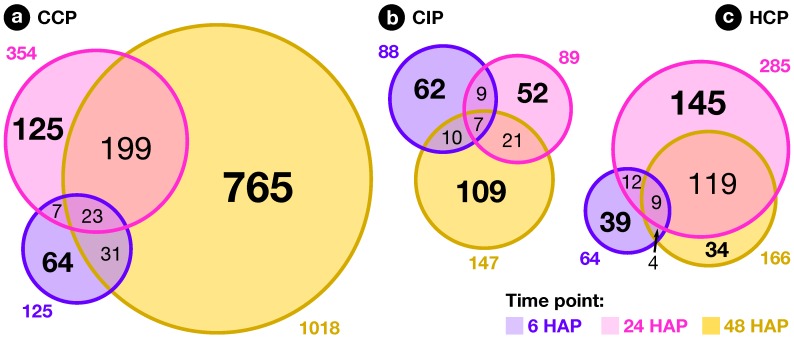
Overlap between time points. Venn diagrams showing the overlap between lists of genes modulated 6, 24 and 48 HAP after CCP, CIP, and HCP.

The exploratory nature of our study led us to opt for relaxed criteria to select regulated genes: Transcripts showing a greater than ±1.5-fold expression difference between test and control hybridizations with P≤0.05 were retained for further analysis (Dataset S1b–h). In the end, 1441 ovary transcripts showed a significant change in abundance in at least one pollination condition, with 163, 598, and 1184 of them regulated 6, 24, and 48 HAP, respectively (Figure 2). To investigate how regulated transcripts behaved across the different conditions under study, we employed a dual approach involving *k*-means hierarchical clustering (Figure 3, Dataset S1i) and Venn diagrams comparing pollinations at each time-point (Figure 4) and vice versa (Figure 5). Statistics about correlations and coregulations between conditions are shown in Figure S1 and Table S1, respectively.

**Figure 6:**
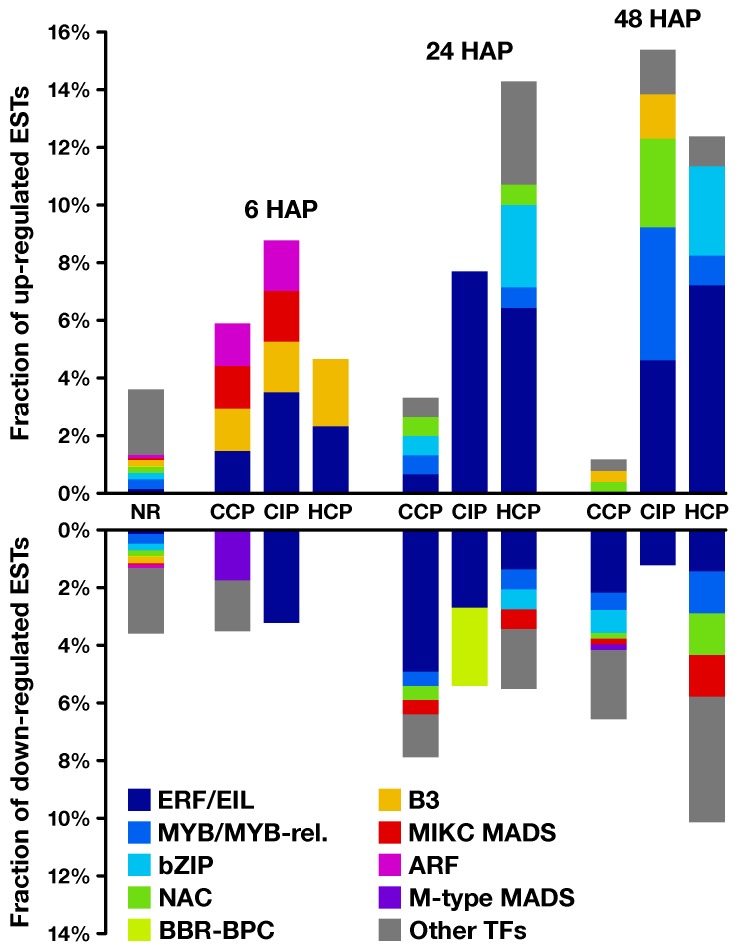
Transcription factor (TF) predictions on regulated genes. For each time point and each pollination condition, proportion of up- (**top**) and down-regulated (**bottom**) genes predicted to encode transcription factors belonging to different families, as identified by the PlantTFDB prediction tool. Corresponding data on non-regulated (*NR*) transcripts is shown as a reference. ARF: auxin response factor; BBR: barley b recombinant; BPC: BASIC PENTACYSTEINE1; bZIP: basic region/leucine zipper motif; EIL: ETHYLENE-INSENSITIVE 3-like; ERF: ethylene-responsive element binding factor; MADS: MCM1, AGAMOUS, DEFICIENS, and SRF; MIKC: MADS-box, intervening, keratin-like, and C-terminal domains; MYB: myeloblastosis virus; NAC: NAM, ATAF, and CUC.

**Figure 7:**
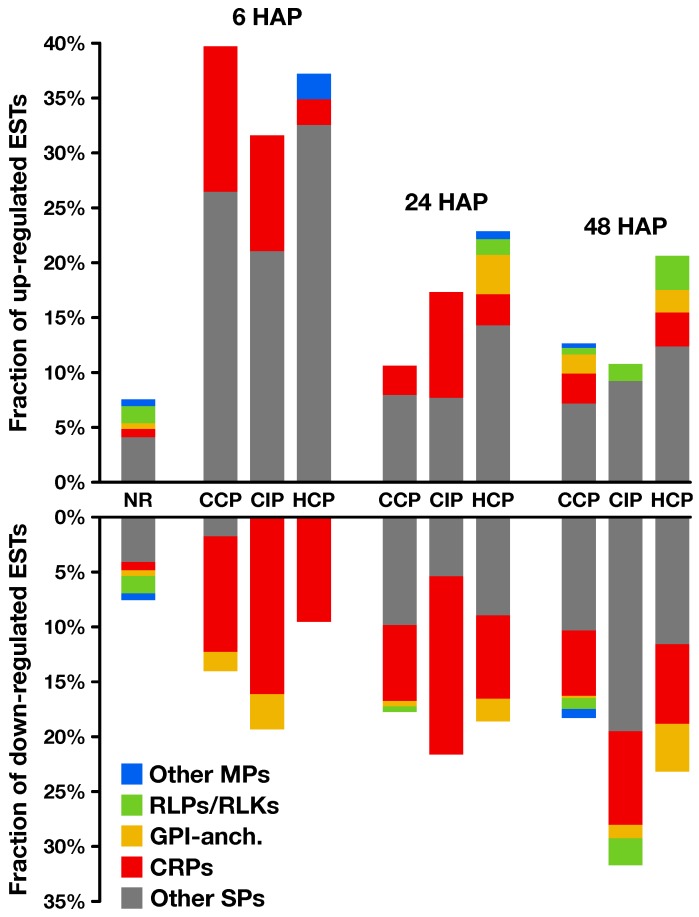
Secretion predictions on regulated genes. For each time point and each pollination condition, proportion of up- (**top**) and down-regulated (**bottom**) genes predicted to encode proteins possessing a secretory signal peptide. Among them, cysteine-rich proteins (*CRPs*, red) and other non-membrane secreted proteins (*Other SPs*, gray), predicted GPI-anchored proteins (*GPI-anch.*, yellow), putative receptor-like proteins and receptor-like kinases (*RLPs/RLKs*, green), and other membrane-bound secreted proteins (*Other MPs*, blue). Corresponding data on non-regulated (*NR*) transcripts is shown as a reference.

Several analyses were performed to better understand the potential functions of regulated transcripts (Dataset S2). First, we proceeded with BLASTn and BLASTx searches against the National Center for Biotechnology Information (NCBI) RefSeq database to find potential homologs in other species and give descriptions to our transcripts (Dataset S3), and then performed a functional classification into GO (*Gene Ontology*) categories and subsequent enrichment analyses (Datasets S4 and S5). Finally, we used the closest BLASTx hit of each EST to perform a variety of *in silico* predictions, in particular putative transcription factors (Figure 6 and Figure S2, Table S2) and secreted proteins (Figure 7 and Figure S3, Table S3), as well as metabolic enzymes (Table S4).

### 2.3. Early Response to Pollination

At 6 HAP, all pollination types had germinated equally and PTs had reached ∼1.5 mm. In all, 163 ovary transcripts showed a statistically significant change in abundance 6 h after CCP, CIP, or HCP (Figure 4a). As can be seen in Figure 2a, the three pollination types induced a globally similar response in the ovary, with significant overlaps between regulated genes (Table S1a–c). Interestingly, pollination responses were also highly correlated to the stigma wounding condition with $R^{2}$ coefficents ranging from 0.80 to 0.93 (Figure S1), but remained clearly distinct from a simple touch response ($R^{2}\leq 0.05$), suggesting that the early response following pollination corresponds to the perception of a wounding aggression, due to PT penetration and growth in the stigma.

Functional categories significantly enriched in common and coregulated transcripts included defense-related GO-terms such as “defense response to fungus” (Dataset S4a–g). Interestingly, at 24 and 48 HAP, this category remained enriched only in transcripts regulated by CIP or HCP, but not CCP (clusters 4 and 18 on Figure 3; Dataset S5d and r), suggesting that the response to CIP and HCP remains more akin to a defense response than CCP at later time points. The GO enrichment analysis also revealed that the non-specific response to pollination 6 HAP was correlated to the modulation of signaling-related categories such as “auxin transport” and “cellular response to reactive oxygen species”. Moreover, transcription factors predicted to belong to ERF and ARF families were also regulated 6 HAP (Figure 6, Table S2a), pointing to a possible involvement of phytohormones in the mediation of early ovary responses to pollination.

In *S. chacoense*, the ovule secretome was shown to consitute a dynamic microenvironnement in preparation for terminal pollen–pistil interactions [60]. Therefore, we investigated the presence of transcripts predicted to encode secreted proteins (SPs) in our dataset (Figure 7, Table S3). Interestingly, they represented 31 to 40% of the transcripts regulated 6 HAP, while they accounted for only 7.5% of non-regulated transcripts, which represents a significant enrichment (Table S3a). An example of SPs induced 6 HAP were xyloglucan endotransglucosylase/hydrolases (XTHs), a group of cell wall-loosening enzymes previously reported to play a role during host invasion by parasitic plant haustoria [61]. Besides XTHs, 15, 11, and 3 cysteine-rich proteins (CRPs) were modulated by CCP, CIP, and HCP, respectively (Figure 7). This peculiar category of small, secreted, rapidly evolving proteins, with ≥6 cysteines and a mature size ≤150 aa, was shown to be involved in several species-specific pollen–pistil interactions [62]. Here, CRPs exhibited a statistically significant enrichment 6 HAP, representing up to 13% of transcripts induced by pollination, and only 0.8% of not regulated transcripts (Table S3a). Interestingly, different CRP families exhibited distinct regulation patterns: lipid-transfer proteins (LTPs) were induced by pollination, while other families such as γ-thionins and metallocarboxypeptidase inhibitors (MCPIs) were repressed. LTPs were previously shown to control PT adhesion and pre-ovular guidance in the pistil [63], while thionin-like proteins were reported to be embryo sac-dependent CRPs with potential roles in PT-ovule interactions [64]. MCPIs, on the other hand, are known to be ovary and fruit development regulators in tomato plants [65]. All this underlines that the early pollination signal participates in the dynamic remodelling of the ovule secretome, affecting proteins susceptible to play key roles for ovary development and functionality.

Finally, even though the three pollination types produced a globally similar response in the ovary 6 HAP, specific profiles already started to be visible, with 52, 22, and 13 transcripts specifically regulated in CCP, CIP, and HCP, respectively (Figure 4a). Moreover, transcripts up-regulated in both conspecific pollinations (CCP and CIP) were specifically enriched in several proteins similar to known regulators of ovule specification and development: ARGONAUTE 4 proteins [66], as well as the AGAMOUS-LIKE 11 [67] and AUXIN RESPONSE FACTOR 5 [68] transcription factors (Dataset S4d). This points to a possible role of conspecific pollination as a signal triggering ovule and female gametophyte development, possibly mediated by ethylene and auxin, as demonstrated previously in other species [38,39,40,41].

### 2.4. Pollination Response after Completion of the SI Reaction

At the second time point, 24 HAP, the majority of CIP PTs had ceased growth, while HCP and CCP PTs had reached around one and two thirds of the style’s length, respectively (Figure 1). Compared to 6 HAP, an amplification of the ovary response to both compatible pollinations was visible, with 354 and 285 transcripts modulated in CCP and HCP, respectively (Figure 4b) and a very limited overlap with early responses (Figure 5a–c and Figure S1). Moreover, a larger proportion of those transcripts became specific to CCP (76% or 269/354) and HCP (69% or 196/285). Even though 58 transcripts appeared in the overlap between CCP and HCP on Figure 4b, the majority of them (57%) had in fact opposite regulations (Table S1d). All this suggests that the two pollination types are now perceived as distinct events by the ovary, as confirmed by the low correlation coefficient on Figure S1 ($R^{2}=0.03$).

This is further supported by the analysis of enriched functional categories. Among them, phytohormone-related GO terms exhibited contrasted responses after CCP and HCP. In particular, categories related to signaling mediated by the diffusible hormone ethylene were enriched in transcripts up-regulated by HCP and down-regulated by CCP (Dataset S4l). GO-terms “response to ethylene” and “ethylene metabolic process” were also significantly over-represented in cluster 4 on Figure 3 (Dataset S5d), while putative ERF/EIL transcription factors shared a consistent enrichment profile (Figure 6, Table S2b), which suggests that ethylene is a key mediator of pollination-specific, long-distance signaling in the pistil. In line with this, previous studies revealed the existence of post-pollination ethylene bursts (PPEBs) occuring in the stigma/style of other solanaceous species such as *Petunia* [69] and tobacco [70,71], whose timing and/or intensity could vary according to the pollination type.

More recently, additional roles were discovered for ethylene signaling in the context of ovule and PT function. While pollination-induced ethylene accumulation in immature tobacco flowers was shown to be correlated to female gametophyte maturation [41], ethylene was also demonstrated to control PT elongation in *Arabidopsis* by affecting the organization of actin microfilaments [72]. Furthermore, the over-accumulation of the ethylene signal-transducing protein ETHYLENE INSENSITIVE 3 (EIN3) in synergid cells was shown to lead to PT attraction defects in *Arabidopsis* [73]. Here, cluster 8, which gathers transcripts specifically down 24 and 48 h after CCP (Figure 3), included two EIN3-like proteins that remained stable after CIP and HCP (Dataset S3d). This suggests that the differential ethylene response in CCP vs. HCP might allow the ovary to get specifically prepared for compatible PT guidance.

Besides ethylene, genes up-regulated in HCP and down-regulated in CCP were also enriched in the GO-term “response to abscisic acid” (Dataset S4l), while “response to gibberellin” was over-represented in cluster 9, consisting of transcripts specifically down in CCP (Dataset S5i). On the other hand, transcripts up-regulated by CCP and/or down-regulated by HCP were enriched in categories related to auxin, brassinosteroids, and jasmonic acid (Dataset S4j and m, Dataset S5r and t). This denotes the existence of a complex cross-talk between phytohormone signaling pathways coordinating the ovary response to pollination.

In contrast, GO-terms associated with the response to reactive oxygen species (ROS) were over-represented in genes specifically up-regulated by CCP (Dataset S4h), as confirmed by the clustering analysis (Figure 3, clusters 14 and 22; Dataset S5n and v). Interestingly, besides being key players of rapid long-distance signaling [74], ROS are known to control pollen germination [75,76] and PT growth [77,78,79]. We could therefore hypothesize that ROS may convey the CCP signal at a distance, or be part of the ovule response to a different CCP signal. Interestingly, ovule-emitted ROS were shown to control PT rupture, with no influence however, on the pollination status [80]. Further work is therefore required to better understand how CCP-induced modulation of ROS-related genes affects ovule function in preparation for interactions restricted to conspecific PTs.

As demonstrated by metabolic pathway (Table S4b) and GO (Dataset S5t) enrichment analyses, enzymes of the secondary metabolism, especially those involved in anthocyanin, flavone, and favonol biosynthesis, were over-represented in transcripts from cluster 20, which were up-regulated by CCP and down-regulated by HCP (Figure 3). Flavonoids, have been extensively studied as messenger molecules during pollination, especially for the control of pollen germination [81]. Interestingly, flavonoids were also shown to play a key role for the maintenance of ROS homeostasis in the context of PT growth [82]. All this suggests that CCP-induced flavonoid production by the ovary could be a mechanism favoring conspecific PT growth in the pistil, in preparation for species-preferential pollen–ovule interactions.

Moreover, cluster 18 (Figure 3) contained a γ-aminobutyric acid (GABA) transaminase, an enzyme responsible for the control of γ-aminobutyric acid (GABA), which was specifically down-regulated after HCP while it remained stable after CCP (Dataset S3d). GABA is a key signaling compound controlling PT elongation [83] and known to form a gradient in the pistil, with increasing concentrations from the stigma to the ovule, whose disruption impairs proper PT directional growth [84]. Therefore, HCP-induced disruption of pistil GABA levels could constitute another mechanism facilitating the rejection of heterospecific pollen.

In contrast to the ample, antagonistic ovary response to CCP and HCP, the number of transcripts regulated by CIP 24 HAP remained stable compared to 6 HAP (89 vs. 88, Figure 4b). Interestingly, transcipts specifically regulated by CIP were enriched in GO-terms such as “gene silencing”, “DNA packaging”, and “chromating remodeling” (Dataset S4i), pointing to a possible epigenetic modulation of gene expression in the ovary as a consequence of self-pollination.

### 2.5. Fertilization and Late Pollination Responses

In *S. chacoense*, as in many *Solanum* species, conspecific fertilization takes place from 36 HAP until 48 HAP as determined by aniline blue staining of the PTs that had reached the ovules (data not shown) and by the fertilization-induced activation of ribosomal proteins [85]. As expected, the highest number of ovule-modulated genes, 1018, were isolated 48 HAP from a fully compatible pollination (CCP) that lead to fertilization, thus including a large number of genes regulated immediately following fertilization (Figure 4c). Among them, 253 (25%) were already regulated before fertilization 6 or 24 HAP, suggesting a dual role for these genes before and after fertilization, during pollination and early embryogenesis (Figure 5a and Table S1g). Remaining genes were particularly present in clusters 5–10 (down-regulation) and 14–16 (up-regulation) and were, as expected, enriched in functional categories related to cell proliferation and gene expression (Dataset S4o, Dataset S5e–j and n–p) underlining that CCP induced the reprogramming of ovule transcriptome toward embryo development.

In contrast, only 147 genes were modulated by CIP. Although only a limited increase was observed in the total number of genes modulated between 24 and 48 HAP in CIP (going up from 89 to 147), the nature of the modulated genes was strikingly different with 74% (109/147) genes specifically expressed 48 HAP (Figure 5b and Table S1h). In fact, as can be seen on Figure 2c, this modulation of the ovule response to CIP 48 HAP still closely resembles a wound response, as confirmed by the high statistical correlation between the two conditions ($R^{2}=0.70$, Figure S1).

Compared to CCP, only 166 genes were modulated in HCP at 48 HAP, an important reduction from 24 HAP (Figure 4c). Among them, no ribosomal protein genes were up-regulated (out of the 65 available on the microarray), indicating that fertilization had yet taken place (Dataset S3c). This was confirmed by aniline blue staining of *S. microdontum* PTs 48 HAP, showing that most of the tubes had only travelled 60% of the style’s length (Figure 1). Interestingly, 77% of the HCP genes regulated at 48 HAP (128 out of 166) were common with the ones expressed 24 HAP, a situation not observed in other pollination types where little overlap was observed between successive time points (Figure 5a–c, Table S1g–i). This is further confirmed by the high statistical correlation between HCP responses 24 and 48 HAP ($R^{2}=0.83$, Figure S1). Importantly, responses to mid-style CCP PTs (24 HAP) and HCP PTs (48 HAP) exhibit a very low correlation coefficient ($R^{2}=0.01$), confirming that the late response to HCP is not simply a non-specific response to PTs located in the central region of the style. In terms of functional annotations, the categories enriched 48 h after HCP mostly overlap those enriched 24 HAP and discussed in the previous section (Datasets S4 and S5). All this shows that HCP is perceived by the ovary as a single, continuous signal, that is clearly distinct from CCP.

## 3. Conclusions

**Figure 8:**
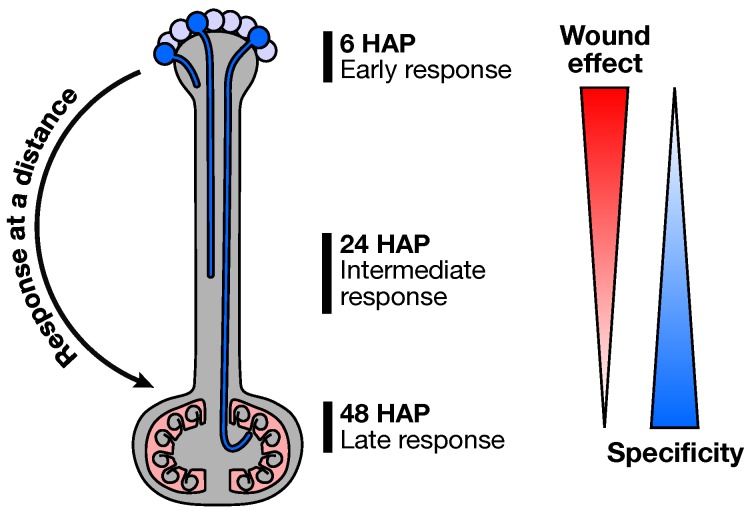
Outline of the at a distance ovary response following pollination. Early pollination response, irrespective of the pollination type, is akin to a wound response. As the PT grow, each pollination type is then recognized as distinct and produce a highly specific transcriptomic signature in the ovary, before PT arrival.

The present study shows that, after being all initially perceived as a wounding aggression, each pollination type produced its own transcriptomic signature at a distance in the ovary, in a way that may prepare subsequent species–preferential pollen–ovule interactions (Figure 8). We have shown that ROS and ethylene are potential messengers acting at a distance to convey the presence of CCP and HCP PTs, respectively. But how could distinct pollination types elicit such antagonistic long-distance responses in the pistil? A recent study revealed that pollination triggers the expression of three pollinic MYB transcription factors, whose mutation causes significant changes in the post-pollination pistil transcriptome. Interestingly, those MYBs were shown to control the expression of rapidly evolving thionin-like CRPs that are secreted by the growing PTs in the pistil, and suspected to control proper PT reception by the ovule [24]. Such divergent PT-secreted proteins could serve as initial signals specific to each pollination type, allowing the pistil to discriminate between CCP, CIP, and HCP PTs.

## 4. Materials and Methods

### 4.1. Plant Material and Pollination Conditions

Self-incompatible *Solanum chacoense* and *S. microdontum* accessions (2n=2x=24) obtained from the NRSP-6 US Potato Genebank (Sturgeon Bay, WI, USA) were glasshouse-grown with a 16 h light/8 h dark cycle. The *S. chacoense* G4 genotype ($S_{12}S_{14}$ SI alleles) was used as the female progenitor. *S. chacoense* pollen from G4 and V22 ($S_{11}S_{13}$) genotypes was used for CIP and CCP, respectively. HCP was performed with pollen from *S. microdontum* PI500041 accession.

Wounding treatments were performed by slightly crushing the upper region of the style with small forceps, as described previously [48]. Touch treatments consisted in mock pollinations accomplished by gently touching the stigmas with sterile 100 μm zirconia/silica beads (BioSpec Products Inc., Bartlesville, OK, USA).

### 4.2. Pollen Tube Growth Assay and Aniline Blue Staining

Flowers were collected from 6 to 96 h after pollination. Pistils were dissected and fixed overnight in FAA (ethanol 50%, water 35% glacial acetic acid 10%, formalin 5%, washed twice with water and then softened in 2 M NaOH for 24 h at room temperature. After rinsing, pistils were stained with 0.1% aniline blue in K_3_PO_4_ buffer (pH 7.5) and slightly squashed between a slide and coverslip. Pictures were taken on a Zeiss Axio Observer.Z1 fluorescence microscope equipped with an AxioCam HRm camera (Carl Zeiss Canada, Canada) and analyzed with ImageJ (https://imagej.nih.gov/ij).

### 4.3. RNA Isolation and Microarray Experimental Design

Ovules were collected 6, 24, and 48 h after each treatment and used for RNA extraction and probe preparation. RNA from UOs served as controls. Four independent biological replicates were produced for each time point. To estimate reproducibility and to produce control data for statistical analyses, a large number of UOs were isolated and separated in seven independent control groups. RNA from randomly selected pairs of controls was hybridized on six microarrays. Microarray experiments and data analysis were performed as described previously [19]. The data discussed in this publication have been deposited in NCBI’s Gene Expression Omnibus (http://www.ncbi.nlm.nih.gov) and are accessible through GEO Series accession number GSE21957 [86].

### 4.4. Differential Expression Analaysis

Transcripts with a significant expression fold-change in CCP, CIP, HCP, W or T vs. UO (≥1.5 or ≤−1.5; P≤0.05, Welch’s *t*-test) were considered to be regulated. To draw the heatmaps (Figure 2), a hierarchical clustering analysis was performed at each time point using genes regulated in at least one pollination condition. Euclidean distances between CCP, CIP, and HCP vs. UO expression ratios were used to connect transcripts based on Ward’s method [87]. Expression ratios in W and T vs. UO were then added to the heatmaps. Clusters presented on Figure 3 were obtained by a similar hierarchical clustering analysis applied to Pearson’s correlation coefficients of UO, CCP, CIP, and HCP vs. UO expression ratios at all time points, based on Ward’s method. Dendrograms were then split into clusters using *k*-means clustering with k=25. Figure S1 presents pairwise squared correlation coefficients ($R^{2}$) of CCP, CIP, HCP, W, and T vs. UO expression values obtained by linear least-squares regression analysis.

### 4.5. Sequence Annotation

ESTs were compared to the NCBI refseq_rna and refseq_protein databases v. 87 [88] using BLASTn and BLASTx v. 2.2.29+, respectively [89]. Descriptions were then manually assigned to each EST based on the most similar hits. Automated functional classification into Gene Ontology (GO) categories was performed with Blast2GO v. 5.2.5 [90]. The best BLASTx hits for each EST were used for transcription factor predictions with the PlantTFDB v. 4.0 prediction tool [91], enzyme code retrieval and metabolic pathway assignment based on the Kyoto Encyclopedia of Genes and Genomes (KEGG) database v. 90.0 [92], and signal peptide predictions with SignalP v. 4.1 [93]. Proteins predicted to contain a signal peptide were further checked for the presence of transmembrane helices (TMH) with TMHMM v. 2.0 [94].

Proteins with one predicted TMH were submitted to HMMER v. 3.1b2 (http://hmmer.org/) to check for the presence of kinase domains (KDs; motifs *Pkinase* and *Pkinase_Tyr*) and leucine-rich repeats (LRRs; motifs *LRR_1*, *LRR_2*, *LRR_4*, *LRR_5*, *LRR_6*, *LRR_8*, *LRR_9*, *LRRNT*, and *LRRNT_2*) defined in the Pfam database v. 32.0 [95]. Among them, those with at least one KD and one LRR were classified as potential LRR receptor-like kinases (LRR-RLKs); those with at least one KD but no LRR were tagged as potential RLKs; remaining proteins with one TMH were considered as potential receptor-like proteins (RLPs). Proteins with two or more predicted TMHs were tagged as other membrane proteins.

Proteins that were not predicted to have a TMH were inspected for the presence of predicted of glycosylphosphatidylinositol (GPI) anchors with the PredGPI program [96]. Remaining proteins were split into cysteine-rich proteins (6 cysteines or more, mature part smaller than 150 aa) and other secreted proteins with KAPPA v. 1.0 [97].

Enrichment analyses based on all those predictions were made using Fisher’s exact tests. A prediction was considered enriched in a given condition when P≤0.05.

## Supplementary Materials

The following are available online at https://www.mdpi.com/2223-7747/8/6/185/s1, Figure S1: Heatmap describing pairwise correlation coefficients of expression ratios between samples, Figure S2: Graphical summary of *in silico* transcription factor predictions made in each cluster, Figure S3: Graphical summary of *in silico* predictions made on secreted proteins in each cluster, Table S1: Number and proportion of genes regulated after each treatment, along with coregulation statistics across conditions and time points, Table S2: Summary of *in silico* transcription factor predictions made on genes modulated at a distance by pollination, with comparative enrichment analyses across conditions and clusters, Table S3: Summary of *in silico* signal peptide and subsequent predictions made on genes modulated at a distance by pollination, with comparative enrichment analyses across conditions and clusters, Table S4: Summary of *in silico* metabolic pathway predictions made on genes modulated at a distance by pollination, with comparative enrichment analyses across conditions and clusters, Dataset S1: Detailed view of microarray data, differential expression analysis, and *k*-means clustering results, Dataset S2: Detailed view of *in silico* annotations made on genes on the microarray: BLAST results, GO functional classification, enzyme and transcription factor predictions, and predictions on secreted proteins, Dataset S3: Detailed view of differential expression data for ovary genes modulated by pollination, Dataset S4: Statistical analysis on GO functional categories enriched in each pollination condition, Dataset S5: Statistical analysis on GO functional categories enriched in each cluster.
