# Supplementary material for: Pollination Type Recognition from a Distance by the Ovary Is Revealed Through a Global Transcriptomic Analysis
Source: Plants (Basel). 2019 Jun 24;8(6):185. doi: 10.3390/plants8060185 (PMC6630372; doi:10.3390/plants8060185)
Supplement: Supplementary file 1 [file plants-08-00185-s001.zip › Joly-2019-Plants_SuppMat_R1/Joly-2019-Plants_Suppl_Fig_Tab.pdf]

Supplementary Figures and Tables

Valentin Joly · Faïza Tebbji · Daniel P. Matton

List of Figures

S1 Heatmap describing pairwise correlation coefficients of expression ratios between samples . . . 2

S2 Graphical summary of *in silico* transcription factor predictions made in each cluster . . . 3

S3 Graphical summary of *in silico* predictions made on secreted proteins in each cluster . . . 4

List of Tables

S1 Number and proportion of genes regulated after each treatment, along with coregulation statistics across conditions and time points. . . . 5

S2 Summary of *in silico* transcription factor predictions made on genes modulated at a distance by pollination, with comparative enrichment analyses across conditions and clusters . . . 14

S3 Summary of *in silico* signal peptide and subsequent predictions made on genes modulated at a distance by pollination, with comparative enrichment analyses across conditions and clusters . . . 17

S4 Summary of *in silico* metabolic pathway predictions made on genes modulated at a distance by pollination, with comparative enrichment analyses across conditions and clusters . . . 19

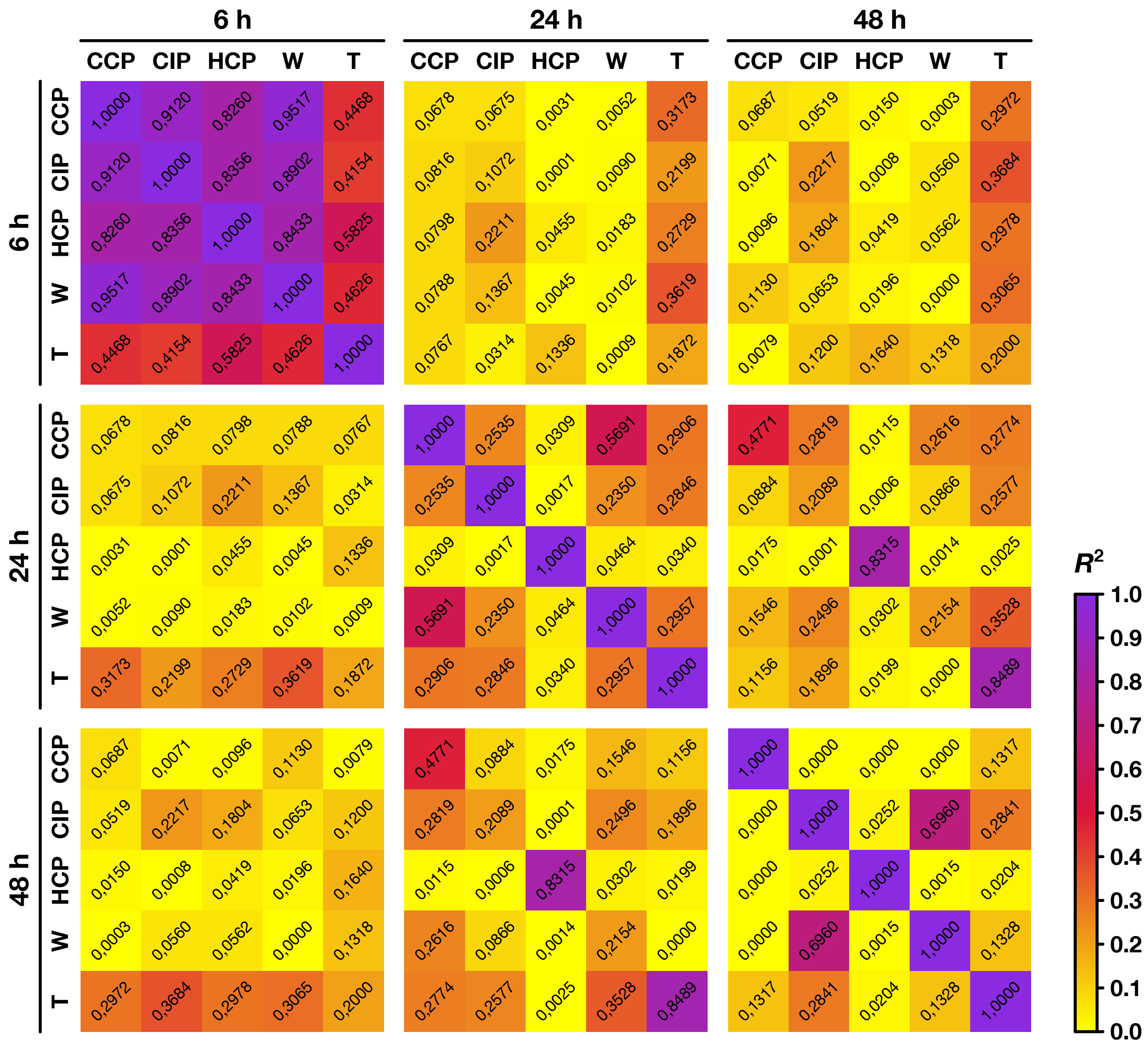

Figure S1. Heatmap describing pairwise correlation coefficients of expression ratios between samples. Squared Pearson's correlation coefficients (R^2) for each pair of samples under study. For two samples X and Y, the linear regression was performed on $\log_2$ values of expression changes (X/UO vs. Y/UO) of genes regulated in X and/or Y.

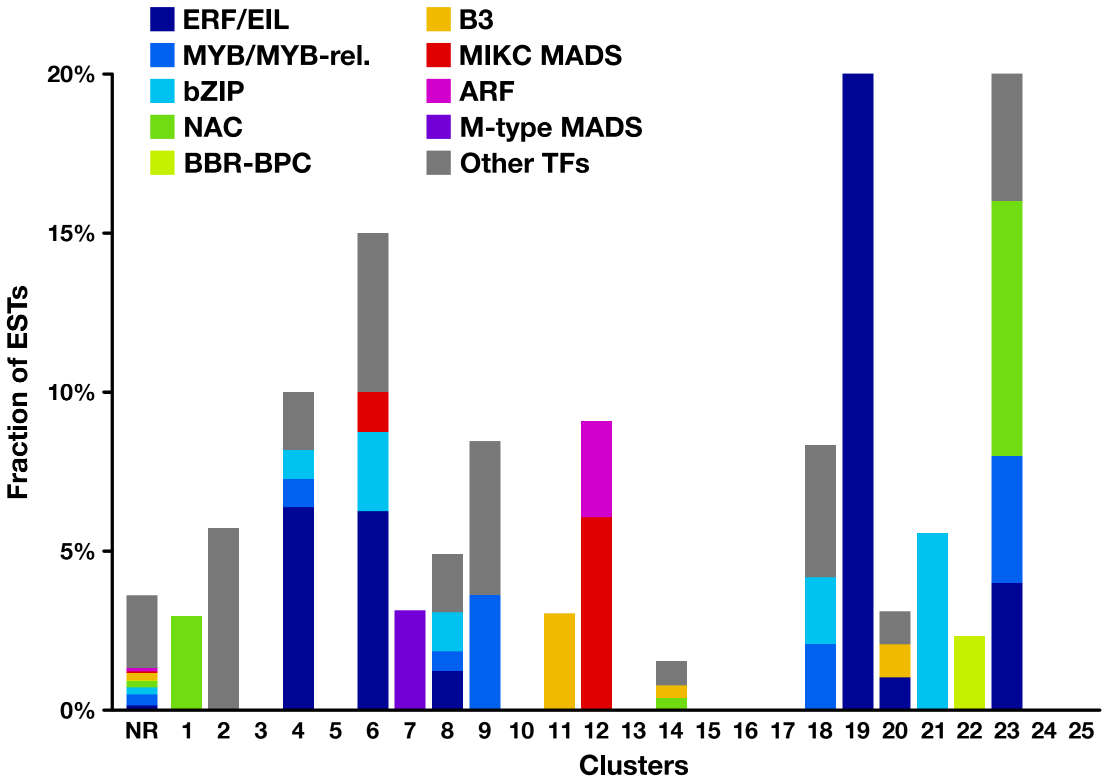

Figure S2. Graphical summary of *in silico* transcription factor predictions made in each cluster. The PlantTFDB prediction tool was used on the best BLASTx hit for each EST to predict and classify sequences into transcription factor (TF) families.

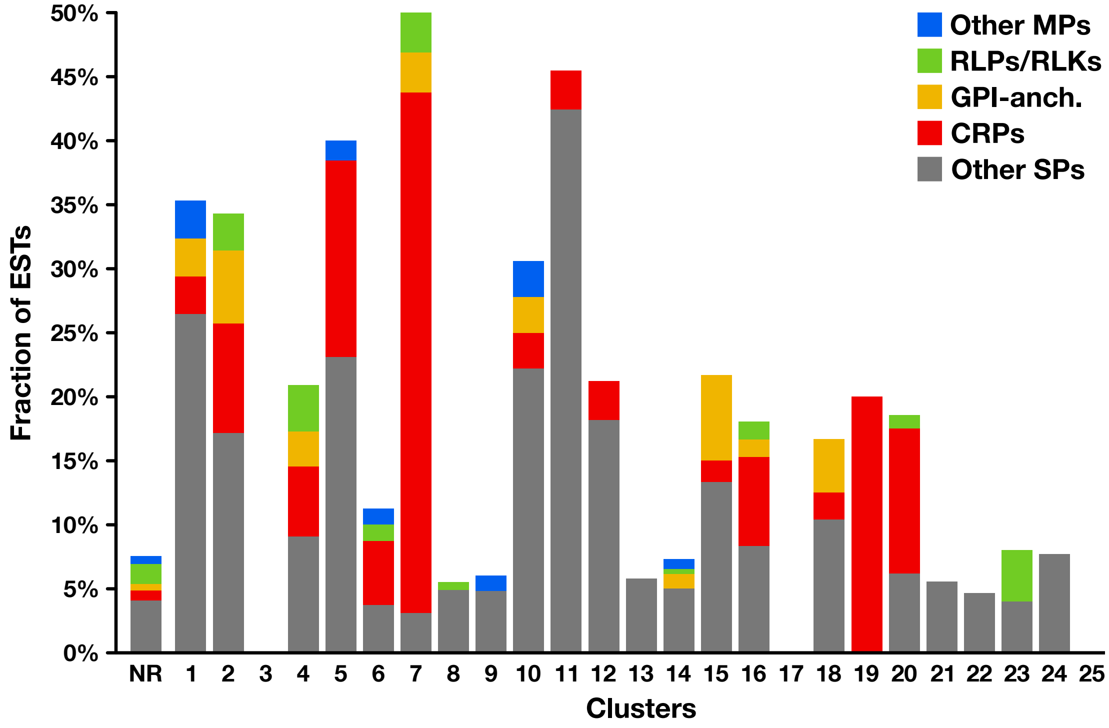

Figure S3. Graphical summary of *in silico* predictions made on secreted proteins in each cluster. SignalP was used on the best BLASTx hit for each EST to predict the presence of a signal peptide. GPI anchors were predicted using PredGPI. Remaining sequences were inspected for the presence of transmembrane helices with TMHMM. Sequences with one such helix were classified as potential receptor-like proteins or kinases (RLPs/RLKs); sequences with multiple transmembrane helices were classified as other membrane proteins. Sequences without a transmembrane helix nor a GPI anchor were split into cysteine-rich proteins (CRPs; mature peptide ≤ 150 aa, 6+ cysteines) and other non-membrane proteins.

Table S1. Number and proportion of genes regulated after each treatment, along with coregulation statistics across conditions and time points.. The number of up- and down-regulated genes in each category/overlap is given in columns "↑" and "↓", respectively. The number of coregulated genes, i.e. having the same regulation in the two conditions compared, is given in columns "↑↑" (up/up) and "↓↓" (down/down). The number of genes in the overlap having opposite regulations are shown in column "Opp.". Values given in column "*p*-values" express the significance of overlaps between gene sets; they were computed with a Fisher's exact test. Local percentages (in black) were computed according to the total number of genes in each row. Global percentages (in gray) were computed according to the total number of genes regulated in the condition. In this table, "specific" refers to genes that are regulated in the considered condition but not in the other pollination conditions at the same time point.

(a) Genes regulated 6 h after conspecific compatible pollination (CCP)

**Category**	**Regulated**			**Coregulated**			**Opp.**	***p*-value**
	**all**	**↑**	**↓**	**↑↑**	**↓↓**	**total**		
**Total**	**125**	**68**	**57**					
	100%	54.4%	45.6%					
	100%	54.4%	45.6%					
**Specific**	**52**	**22**	**30**					
	100%	42.3%	57.7%					
	41.6%	17.6%	24.0%					
**Non specific**	**73**	**46**	**27**					
	100%	63.0%	37.0%					
	58.4%	36.8%	21.6%					
**Common to CIP 6 h**	**63**	**40**	**23**	**40**	**23**	**63**	**0**	1.2×10^{-94}
	100%	63.5%	36.5%	63.5%	36.5%	100%	0.0%	
	50.4%	32.0%	18.4%	32.0%	18.4%	50.4%	0.0%	
**Common to HCP 6 h**	**48**	**30**	**18**	**30**	**18**	**48**	**0**	7.8×10^{-130}
	100%	62.5%	37.5%	62.5%	37.5%	100%	0.0%	
	38.4%	24.0%	14.4%	24.0%	14.4%	38.4%	0.0%	
**Common to W 6 h**	**90**	**49**	**41**	**49**	**41**	**90**	**0**	1.0×10^{-42}
	100%	54.4%	45.6%	54.4%	45.6%	100%	0.0%	
	72.0%	39.2%	32.8%	39.2%	32.8%	72.0%	0.0%	
**Common to T 6 h**	**55**	**35**	**20**	**35**	**18**	**53**	**2**	1.0×10^{-90}
	100%	63.6%	36.4%	63.6%	32.7%	96.4%	3.6%	
	44.0%	28.0%	16.0%	28.0%	14.4%	42.4%	1.6%	
**Common to CCP 24 h**	**30**	**16**	**14**	**11**	**11**	**22**	**8**	4.0×10^{-92}
	100%	53.3%	46.7%	36.7%	36.7%	73.3%	26.7%	
	24.0%	12.8%	11.2%	8.8%	8.8%	17.6%	6.4%	
**Common to CCP 48 h**	**54**	**32**	**22**	**16**	**20**	**36**	**18**	3.3×10^{-27}
	100%	59.3%	40.7%	29.6%	37.0%	66.7%	33.3%	
	43.2%	25.6%	17.6%	12.8%	16.0%	28.8%	14.4%	

Table S1. (continued)

(b) Genes regulated 6 h after conspecific incompatible pollination (CIP)

**Category**	**Regulated**			**Coregulated**			**Opp.**	***p*-value**
	**all**	↑	↓	↑↑	↓↓	**total**		
**Total**	**88**	**57**	**31**					
	100%	64.8%	35.2%					
	100%	64.8%	35.2%					
**Specific**	**22**	**15**	**7**					
	100%	68.2%	31.8%					
	25.0%	17.0%	8.0%					
**Non specific**	**66**	**42**	**24**					
	100%	63.6%	36.4%					
	75.0%	47.7%	27.3%					
**Common to CCP 6 h**	**63**	**40**	**23**	**40**	**23**	**63**	**0**	4.0×10^{-28}
	100%	63.5%	36.5%	63.5%	36.5%	100%	0.0%	
	71.6%	45.5%	26.1%	45.5%	26.1%	71.6%	0.0%	
**Common to HCP 6 h**	**41**	**26**	**15**	**26**	**15**	**41**	**0**	8.6×10^{-78}
	100%	63.4%	36.6%	63.4%	36.6%	100%	0.0%	
	46.6%	29.5%	17.0%	29.5%	17.0%	46.6%	0.0%	
**Common to W 6 h**	**63**	**39**	**24**	**39**	**24**	**63**	**0**	1.4×10^{-27}
	100%	61.9%	38.1%	61.9%	38.1%	100%	0.0%	
	71.6%	44.3%	27.3%	44.3%	27.3%	71.6%	0.0%	
**Common to T 6 h**	**46**	**31**	**15**	**31**	**14**	**45**	**1**	9.1×10^{-52}
	100%	67.4%	32.6%	67.4%	30.4%	97.8%	2.2%	
	52.3%	35.2%	17.0%	35.2%	15.9%	51.1%	1.1%	
**Common to CIP 24 h**	**16**	**10**	**6**	**9**	**6**	**15**	**1**	4.5×10^{-113}
	100%	62.5%	37.5%	56.2%	37.5%	93.8%	6.2%	
	18.2%	11.4%	6.8%	10.2%	6.8%	17.0%	1.1%	
**Common to CIP 48 h**	**17**	**14**	**3**	**13**	**2**	**15**	**2**	1.5×10^{-97}
	100%	82.4%	17.6%	76.5%	11.8%	88.2%	11.8%	
	19.3%	15.9%	3.4%	14.8%	2.3%	17.0%	2.3%	

Table S1. (continued)

(c) Genes regulated 6 h after heterospecific compatible pollination (HCP)

Category	Regulated			Coregulated			Opp.	*p*-value
	all	↑	↓	↑↑	↓↓	total		
**Total**	**64**	**43**	**21**					
	100%	67.2%	32.8%					
	100%	67.2%	32.8%					
**Specific**	**13**	**11**	**2**					
	100%	84.6%	15.4%					
	20.3%	17.2%	3.1%					
**Non specific**	**51**	**32**	**19**					
	100%	62.7%	37.3%					
	79.7%	50.0%	29.7%					
**Common to CCP 6 h**	**48**	**30**	**18**	**30**	**18**	**48**	**0**	1.2×10^{-16}
	100%	62.5%	37.5%	62.5%	37.5%	100%	0.0%	
	75.0%	46.9%	28.1%	46.9%	28.1%	75.0%	0.0%	
**Common to CIP 6 h**	**41**	**26**	**15**	**26**	**15**	**41**	**0**	4.1×10^{-31}
	100%	63.4%	36.6%	63.4%	36.6%	100%	0.0%	
	64.1%	40.6%	23.4%	40.6%	23.4%	64.1%	0.0%	
**Common to W 6 h**	**49**	**30**	**19**	**30**	**19**	**49**	**0**	4.6×10^{-15}
	100%	61.2%	38.8%	61.2%	38.8%	100%	0.0%	
	76.6%	46.9%	29.7%	46.9%	29.7%	76.6%	0.0%	
**Common to T 6 h**	**43**	**31**	**12**	**31**	**12**	**43**	**0**	4.4×10^{-23}
	100%	72.1%	27.9%	72.1%	27.9%	100%	0.0%	
	67.2%	48.4%	18.8%	48.4%	18.8%	67.2%	0.0%	
**Common to HCP 24 h**	**21**	**15**	**6**	**13**	**3**	**16**	**5**	1.5×10^{-43}
	100%	71.4%	28.6%	61.9%	14.3%	76.2%	23.8%	
	32.8%	23.4%	9.4%	20.3%	4.7%	25.0%	7.8%	
**Common to HCP 48 h**	**13**	**10**	**3**	**7**	**2**	**9**	**4**	3.1×10^{-68}
	100%	76.9%	23.1%	53.8%	15.4%	69.2%	30.8%	
	20.3%	15.6%	4.7%	10.9%	3.1%	14.1%	6.2%	

Table S1. (continued)

(d) Genes regulated 24 h after conspecific compatible pollination (CCP)

**Category**	**Regulated**			**Coregulated**			**Opp.**	***p*-value**
	**all**	**↑**	**↓**	**↑↑**	**↓↓**	**total**		
**Total**	**354**	**151**	**203**					
	100%	42.7%	57.3%					
	100%	42.7%	57.3%					
**Specific**	**269**	**128**	**141**					
	100%	47.6%	52.4%					
	76.0%	36.2%	39.8%					
**Non specific**	**85**	**23**	**62**					
	100%	27.1%	72.9%					
	24.0%	6.5%	17.5%					
**Common to CIP 24 h**	**41**	**14**	**27**	**14**	**23**	**37**	**4**	0
	100%	34.1%	65.9%	34.1%	56.1%	90.2%	9.8%	
	11.6%	4.0%	7.6%	4.0%	6.5%	10.5%	1.1%	
**Common to HCP 24 h**	**58**	**14**	**44**	**8**	**17**	**25**	**33**	6.1×10^{-307}
	100%	24.1%	75.9%	13.8%	29.3%	43.1%	56.9%	
	16.4%	4.0%	12.4%	2.3%	4.8%	7.1%	9.3%	
**Common to W 24 h**	**61**	**24**	**37**	**24**	**37**	**61**	**0**	0
	100%	39.3%	60.7%	39.3%	60.7%	100%	0.0%	
	17.2%	6.8%	10.5%	6.8%	10.5%	17.2%	0.0%	
**Common to T 24 h**	**56**	**30**	**26**	**30**	**23**	**53**	**3**	0
	100%	53.6%	46.4%	53.6%	41.1%	94.6%	5.4%	
	15.8%	8.5%	7.3%	8.5%	6.5%	15.0%	0.8%	
**Common to CCP 6 h**	**30**	**14**	**16**	**11**	**11**	**22**	**8**	0
	100%	46.7%	53.3%	36.7%	36.7%	73.3%	26.7%	
	8.5%	4.0%	4.5%	3.1%	3.1%	6.2%	2.3%	
**Common to CCP 48 h**	**222**	**97**	**125**	**95**	**122**	**217**	**5**	1.2×10^{-30}
	100%	43.7%	56.3%	42.8%	55.0%	97.7%	2.3%	
	62.7%	27.4%	35.3%	26.8%	34.5%	61.3%	1.4%	

Table S1. (continued)

(e) Genes regulated 24 h after conspecific incompatible pollination (CIP)

Category	Regulated			Coregulated			Opp.	*p*-value
	all	↑	↓	↑↑	↓↓	total		
**Total**	**89**	**52**	**37**					
	100%	58.4%	41.6%					
	100%	58.4%	41.6%					
**Specific**	**17**	**9**	**8**					
	100%	52.9%	47.1%					
	19.1%	10.1%	9.0%					
**Non specific**	**72**	**43**	**29**					
	100%	59.7%	40.3%					
	80.9%	48.3%	32.6%					
**Common to CCP 24 h**	**41**	**18**	**23**	**14**	**23**	**37**	**4**	1.9×10^{-38}
	100%	43.9%	56.1%	34.1%	56.1%	90.2%	9.8%	
	46.1%	20.2%	25.8%	15.7%	25.8%	41.6%	4.5%	
**Common to HCP 24 h**	**45**	**34**	**11**	**15**	**6**	**21**	**24**	8.4×10^{-38}
	100%	75.6%	24.4%	33.3%	13.3%	46.7%	53.3%	
	50.6%	38.2%	12.4%	16.9%	6.7%	23.6%	27.0%	
**Common to W 24 h**	**33**	**24**	**9**	**23**	**9**	**32**	**1**	6.6×10^{-78}
	100%	72.7%	27.3%	69.7%	27.3%	97.0%	3.0%	
	37.1%	27.0%	10.1%	25.8%	10.1%	36.0%	1.1%	
**Common to T 24 h**	**30**	**24**	**6**	**24**	**5**	**29**	**1**	2.4×10^{-70}
	100%	80.0%	20.0%	80.0%	16.7%	96.7%	3.3%	
	33.7%	27.0%	6.7%	27.0%	5.6%	32.6%	1.1%	
**Common to CIP 6 h**	**16**	**9**	**7**	**9**	**6**	**15**	**1**	6.0×10^{-115}
	100%	56.2%	43.8%	56.2%	37.5%	93.8%	6.2%	
	18.0%	10.1%	7.9%	10.1%	6.7%	16.9%	1.1%	
**Common to CIP 48 h**	**28**	**10**	**18**	**9**	**18**	**27**	**1**	6.0×10^{-79}
	100%	35.7%	64.3%	32.1%	64.3%	96.4%	3.6%	
	31.5%	11.2%	20.2%	10.1%	20.2%	30.3%	1.1%	

Table S1. (continued)

(f) Genes regulated 24 h after heterospecific compatible pollination (HCP)

Category	Regulated			Coregulated			Opp.	*p*-value
	all	↑	↓	↑↑	↓↓	total		
**Total**	**285**	**140**	**145**					
	100%	49.1%	50.9%					
	100%	49.1%	50.9%					
**Specific**	**196**	**93**	**103**					
	100%	47.4%	52.6%					
	68.8%	32.6%	36.1%					
**Non specific**	**89**	**47**	**42**					
	100%	52.8%	47.2%					
	31.2%	16.5%	14.7%					
**Common to CCP 24 h**	**58**	**35**	**23**	**8**	**17**	**25**	**33**	8.1×10^{-217}
	100%	60.3%	39.7%	13.8%	29.3%	43.1%	56.9%	
	20.4%	12.3%	8.1%	2.8%	6.0%	8.8%	11.6%	
**Common to CIP 24 h**	**45**	**20**	**25**	**15**	**6**	**21**	**24**	0
	100%	44.4%	55.6%	33.3%	13.3%	46.7%	53.3%	
	15.8%	7.0%	8.8%	5.3%	2.1%	7.4%	8.4%	
**Common to W 24 h**	**37**	**10**	**27**	**5**	**5**	**10**	**27**	0
	100%	27.0%	73.0%	13.5%	13.5%	27.0%	73.0%	
	13.0%	3.5%	9.5%	1.8%	1.8%	3.5%	9.5%	
**Common to T 24 h**	**41**	**4**	**37**	**4**	**9**	**13**	**28**	1.8×10^{-298}
	100%	9.8%	90.2%	9.8%	22.0%	31.7%	68.3%	
	14.4%	1.4%	13.0%	1.4%	3.2%	4.6%	9.8%	
**Common to HCP 6 h**	**21**	**16**	**5**	**13**	**3**	**16**	**5**	0
	100%	76.2%	23.8%	61.9%	14.3%	76.2%	23.8%	
	7.4%	5.6%	1.8%	4.6%	1.1%	5.6%	1.8%	
**Common to HCP 48 h**	**128**	**70**	**58**	**70**	**58**	**128**	**0**	3.0×10^{-199}
	100%	54.7%	45.3%	54.7%	45.3%	100%	0.0%	
	44.9%	24.6%	20.4%	24.6%	20.4%	44.9%	0.0%	

Table S1. (continued)

(g) Genes regulated 48 h after conspecific compatible pollination (CCP)

Category	Regulated			Coregulated			Opp.	*p*-value
	all	↑	↓	↑↑	↓↓	total		
**Total**	**1018**	**515**	**503**					
	100%	50.6%	49.4%					
	100%	50.6%	49.4%					
**Specific**	**893**	**464**	**429**					
	100%	52.0%	48.0%					
	87.7%	45.6%	42.1%					
**Non specific**	**125**	**51**	**74**					
	100%	40.8%	59.2%					
	12.3%	5.0%	7.3%					
**Common to CIP 48 h**	**76**	**35**	**41**	**14**	**28**	**42**	**34**	0
	100%	46.1%	53.9%	18.4%	36.8%	55.3%	44.7%	
	7.5%	3.4%	4.0%	1.4%	2.8%	4.1%	3.3%	
**Common to HCP 48 h**	**60**	**24**	**36**	**13**	**11**	**24**	**36**	0
	100%	40.0%	60.0%	21.7%	18.3%	40.0%	60.0%	
	5.9%	2.4%	3.5%	1.3%	1.1%	2.4%	3.5%	
**Common to W 48 h**	**80**	**43**	**37**	**29**	**24**	**53**	**27**	0
	100%	53.8%	46.2%	36.2%	30.0%	66.2%	33.8%	
	7.9%	4.2%	3.6%	2.8%	2.4%	5.2%	2.7%	
**Common to T 48 h**	**43**	**16**	**27**	**16**	**21**	**37**	**6**	0
	100%	37.2%	62.8%	37.2%	48.8%	86.0%	14.0%	
	4.2%	1.6%	2.7%	1.6%	2.1%	3.6%	0.6%	
**Common to CCP 6 h**	**54**	**18**	**36**	**16**	**20**	**36**	**18**	0
	100%	33.3%	66.7%	29.6%	37.0%	66.7%	33.3%	
	5.3%	1.8%	3.5%	1.6%	2.0%	3.5%	1.8%	
**Common to CCP 24 h**	**222**	**98**	**124**	**95**	**122**	**217**	**5**	0
	100%	44.1%	55.9%	42.8%	55.0%	97.7%	2.3%	
	21.8%	9.6%	12.2%	9.3%	12.0%	21.3%	0.5%	

Table S1. (continued)

(h) Genes regulated 48 h after conspecific incompatible pollination (CIP)

Category	Regulated			Coregulated			Opp.	*p*-value
	all	↑	↓	↑↑	↓↓	total		
**Total**	**147**	**65**	**82**					
	100%	44.2%	55.8%					
	100%	44.2%	55.8%					
**Specific**	**60**	**33**	**27**					
	100%	55.0%	45.0%					
	40.8%	22.4%	18.4%					
**Non specific**	**87**	**32**	**55**					
	100%	36.8%	63.2%					
	59.2%	21.8%	37.4%					
**Common to CCP 48 h**	**76**	**27**	**49**	**14**	**28**	**42**	**34**	3.4×10^{-22}
	100%	35.5%	64.5%	18.4%	36.8%	55.3%	44.7%	
	51.7%	18.4%	33.3%	9.5%	19.0%	28.6%	23.1%	
**Common to HCP 48 h**	**22**	**14**	**8**	**8**	**0**	**8**	**14**	8.7×10^{-165}
	100%	63.6%	36.4%	36.4%	0.0%	36.4%	63.6%	
	15.0%	9.5%	5.4%	5.4%	0.0%	5.4%	9.5%	
**Common to W 48 h**	**77**	**40**	**37**	**40**	**36**	**76**	**1**	2.0×10^{-75}
	100%	51.9%	48.1%	51.9%	46.8%	98.7%	1.3%	
	52.4%	27.2%	25.2%	27.2%	24.5%	51.7%	0.7%	
**Common to T 48 h**	**19**	**12**	**7**	**12**	**6**	**18**	**1**	2.3×10^{-197}
	100%	63.2%	36.8%	63.2%	31.6%	94.7%	5.3%	
	12.9%	8.2%	4.8%	8.2%	4.1%	12.2%	0.7%	
**Common to CIP 6 h**	**17**	**14**	**3**	**13**	**2**	**15**	**2**	3.5×10^{-201}
	100%	82.4%	17.6%	76.5%	11.8%	88.2%	11.8%	
	11.6%	9.5%	2.0%	8.8%	1.4%	10.2%	1.4%	
**Common to CIP 24 h**	**28**	**9**	**19**	**9**	**18**	**27**	**1**	1.1×10^{-180}
	100%	32.1%	67.9%	32.1%	64.3%	96.4%	3.6%	
	19.0%	6.1%	12.9%	6.1%	12.2%	18.4%	0.7%	

Table S1. (continued)

(i) Genes regulated 48 h after heterospecific compatible pollination (HCP)

Category	Regulated			Coregulated			Opp.	*p*-value
	all	↑	↓	↑↑	↓↓	total		
**Total**	**166**	**97**	**69**					
	100%	58.4%	41.6%					
	100%	58.4%	41.6%					
**Specific**	**95**	**51**	**44**					
	100%	53.7%	46.3%					
	57.2%	30.7%	26.5%					
**Non specific**	**71**	**46**	**25**					
	100%	64.8%	35.2%					
	42.8%	27.7%	15.1%					
**Common to CCP 48 h**	**60**	**38**	**22**	**13**	**11**	**24**	**36**	5.1×10^{-46}
	100%	63.3%	36.7%	21.7%	18.3%	40.0%	60.0%	
	36.1%	22.9%	13.3%	7.8%	6.6%	14.5%	21.7%	
**Common to CIP 48 h**	**22**	**16**	**6**	**8**	**0**	**8**	**14**	1.1×10^{-195}
	100%	72.7%	27.3%	36.4%	0.0%	36.4%	63.6%	
	13.3%	9.6%	3.6%	4.8%	0.0%	4.8%	8.4%	
**Common to W 48 h**	**22**	**15**	**7**	**12**	**0**	**12**	**10**	5.2×10^{-184}
	100%	68.2%	31.8%	54.5%	0.0%	54.5%	45.5%	
	13.3%	9.0%	4.2%	7.2%	0.0%	7.2%	6.0%	
**Common to T 48 h**	**13**	**2**	**11**	**2**	**1**	**3**	**10**	1.3×10^{-236}
	100%	15.4%	84.6%	15.4%	7.7%	23.1%	76.9%	
	7.8%	1.2%	6.6%	1.2%	0.6%	1.8%	6.0%	
**Common to HCP 6 h**	**13**	**8**	**5**	**7**	**2**	**9**	**4**	2.0×10^{-249}
	100%	61.5%	38.5%	53.8%	15.4%	69.2%	30.8%	
	7.8%	4.8%	3.0%	4.2%	1.2%	5.4%	2.4%	
**Common to HCP 24 h**	**128**	**70**	**58**	**70**	**58**	**128**	**0**	1.5×10^{-24}
	100%	54.7%	45.3%	54.7%	45.3%	100%	0.0%	
	77.1%	42.2%	34.9%	42.2%	34.9%	77.1%	0.0%	

Table S2. Summary of *in silico* transcription factor predictions made on genes modulated at a distance by pollination, with comparative enrichment analyses across conditions and clusters. The PlantTFDB prediction tool was used on the best BLASTx hit for each EST to predict and classify sequences into transcription factor (TF) families. Columns *Poll.* and *Rest* give the total number of genes regulated in at least one pollination condition, and the number of remaining genes, respectively. Asterisks (*) indicate a significant enrichment for a given prediction in a given sample (Fisher's exact test, $P < 0.05$.

(a) Genes regulated 6 h after pollination

**Prediction**	**CCP 6 h**		**CIP 6 h**		**HCP 6 h**		**Poll.**	**Rest**
	↑	↓	↑	↓	↑	↓		
ERF/EIL	1		*2	1	1		*17	8
MYB/MYB-rel.							7	18
bZIP							7	12
NAC							4	11
BBR-BPC							1	1
B3	1		1		1		3	12
MIKC MADS	1		*1				3	3
ARF	1		1				1	6
M-type MADS		*1					1	
Other TFs		1					21	121
Total TFs	4	2	5	1	2		65	192
**Total**	**68**	**57**	**57**	**31**	**43**	**21**	**1441**	**5340**

(b) Genes regulated 24 h after pollination

**Prediction**	**CCP 24 h**		**CIP 24 h**		**HCP 24 h**		**Poll.**	**Rest**
	↑	↓	↑	↓	↑	↓		
ERF/EIL	1	*10	*4	1	*9	2	*17	8
MYB/MYB-rel.	1	1			1	1	7	18
bZIP	1				*4	1	7	12
NAC	1	1			1		4	11
BBR-BPC				*1			1	1
B3							3	12
MIKC MADS		1				1	3	3
ARF							1	6
M-type MADS							1	
Other TFs	1	3			5	3	21	121
Total TFs	5	*16	4	2	*20	8	65	192
**Total**	**151**	**203**	**52**	**37**	**140**	**145**	**1441**	**5340**

Table S2. (continued)

(c) Genes regulated 48 h after pollination

**Prediction**	**CCP 48 h**		**CIP 48 h**		**HCP 48 h**		**Poll.**	**Rest**
	↑	↓	↑	↓	↑	↓		
ERF/EIL		*11	*3	1	*7	1	*17	8
MYB/MYB-rel.		3	*3		1	1	7	18
bZIP		*4			*3		7	12
NAC	2	1	*2			1	4	11
BBR-BPC							1	1
B3	2		1				3	12
MIKC MADS		1				1	3	3
ARF							1	6
M-type MADS		1					1	
Other TFs	2	12	1		1	3	21	121
Total TFs	6	*33	*10	1	*12	*7	65	192
**Total**	**515**	**503**	**65**	**82**	**97**	**69**	**1441**	**5340**

(d) Pollination-induced genes belonging to clusters 1–12

**Prediction**	**Clusters**												**Poll.**	**Rest**
	**1**	**2**	**3**	**4**	**5**	**6**	**7**	**8**	**9**	**10**	**11**	**12**		
ERF/EIL				*7		*5		2					*17	8
MYB/MYB-rel.				1				1	*3				7	18
bZIP				1		*2		2					7	12
NAC	1												4	11
BBR-BPC													1	1
B3											1		3	12
MIKC MADS						1						*2	3	3
ARF												*1	1	6
M-type MADS							*1						1	
Other TFs		2		2		4		3	4				21	121
Total TFs	1	2		*11		*12	1	8	*7		1	3	65	192
**Total**	**34**	**35**	**15**	**110**	**65**	**80**	**32**	**163**	**83**	**36**	**33**	**33**	**1441**	**5340**

Table S2. (continued)

(e) Pollination-induced genes belonging to clusters 13–25

**Prediction**	**Clusters**													**Poll.**	**Rest**
	**13**	**14**	**15**	**16**	**17**	**18**	**19**	**20**	**21**	**22**	**23**	**24**	**25**		
ERF/EIL							*1	1			1			*17	8
MYB/MYB-rel.						1					1			7	18
bZIP						1			*1					7	12
NAC		1									*2			4	11
BBR-BPC										*1				1	1
B3		1						1						3	12
MIKC MADS														3	3
ARF														1	6
M-type MADS														1	
Other TFs		2				2		1			1			21	121
Total TFs		4				4	1	3	1	1	*5			65	192
**Total**	**52**	**260**	**60**	**72**	**10**	**48**	**5**	**97**	**18**	**43**	**25**	**13**	**19**	**1441**	**5340**

Table S3. Summary of *in silico* signal peptide and subsequent predictions made on genes modulated at a distance by pollination, with comparative enrichment analyses across conditions and clusters. SignalP was used on the best BLASTx hit for each EST to predict the presence of a signal peptide. GPI anchors were predicted using PredGPI. Remaining sequences were inspected for the presence of transmembrane helices with TMHMM. Sequences with one such helix were classified as potential receptor-like proteins or kinases (RLPs/RLKs); sequences with multiple transmembrane helices were classified as other membrane proteins. Sequences without a transmembrane helix nor a GPI anchor were split into cysteine-rich proteins (CRPs; mature peptide ≤ 150 aa, 6+ cysteines) and other non-membrane proteins. Columns *Poll.* and *Rest* give the total number of genes regulated in at least one pollination condition, and the number of remaining genes, respectively. Asterisks (*) indicate a significant enrichment for a given prediction in a given sample (Fisher's exact test, $P < 0.05$).

(a) Genes regulated 6 h after pollination

**Prediction**	**CCP 6 h**		**CIP 6 h**		**HCP 6 h**		**Poll.**	**Rest**
	↑	↓	↑	↓	↑	↓		
CRPs	*9	*6	*6	*5	1	*2	*59	40
Other non-membrane	*18	1	*12		*14		*130	219
GPI-anchored		1		1			*18	28
RLPs/RLKs							12	*84
Other membrane					1		7	32
Total secreted	*27	8	*18	6	*16	2	*226	403
**Total**	**68**	**57**	**57**	**31**	**43**	**21**	**1441**	**5340**

(b) Genes regulated 24 h after pollination

**Prediction**	**CCP 24 h**		**CIP 24 h**		**HCP 24 h**		**Poll.**	**Rest**
	↑	↓	↑	↓	↑	↓		
CRPs	4	*14	*5	*6	4	*11	*59	40
Other non-membrane	12	*20	4	2	*20	13	*130	219
GPI-anchored		1			*5	3	*18	28
RLPs/RLKs		1			2		12	*84
Other membrane					1		7	32
Total secreted	16	*36	9	*8	*32	*27	*226	403
**Total**	**151**	**203**	**52**	**37**	**140**	**145**	**1441**	**5340**

Table S3. (continued)

(c) Genes regulated 48 h after pollination

Prediction	CCP 48 h		CIP 48 h		HCP 48 h		Poll.	Rest
	↑	↓	↑	↓	↑	↓		
CRPs	*14	*30		*7	3	*5	*59	40
Other non-membrane	*37	*52	6	*16	*12	*8	*130	219
GPI-anchored	*9	1		1	2	*3	*18	28
RLPs/RLKs	3	5	1	2	3		12	*84
Other membrane	2	4					7	32
Total secreted	*65	*92	7	*26	*20	*16	*226	403
**Total**	**515**	**503**	**65**	**82**	**97**	**69**	**1441**	**5340**

(d) Pollination-induced genes belonging to clusters 1–12

Prediction	Clusters												Poll.	Rest
	1	2	3	4	5	6	7	8	9	10	11	12		
CRPs	1	*3		*6	*10	*4	*13			1	1	1	*59	40
Other non-mb	*9	*6		10	*15	3	1	8	4	*8	*14	*6	*130	219
GPI-anchored	1	*2		*3			1			1			*18	28
RLPs/RLKs		1		4		1	1	1					12	*84
Other mb	1				1	1			1	1			7	32
Total secreted	*12	*12		*23	*26	9	*16	9	5	*11	*15	*7	*226	403
**Total**	**34**	**35**	**15**	**110**	**65**	**80**	**32**	**163**	**83**	**36**	**33**	**33**	**1441**	**5340**

(e) Pollination-induced genes belonging to clusters 13–25

Prediction	Clusters													Poll.	Rest
	13	14	15	16	17	18	19	20	21	22	23	24	25		
CRPs			1	*5		1	1	*11						*59	40
Other non-mb	3	13	*8	6		5		6	1	2	1	1		*130	219
GPI-anchored		3	*4	1		*2								*18	28
RLPs/RLKs		1		1				1			1			12	*84
Other mb		2												7	32
Total secreted	3	19	*13	*13		8	1	*18	1	2	2	1		*226	403
**Total**	**52**	**260**	**60**	**72**	**10**	**48**	**5**	**97**	**18**	**43**	**25**	**13**	**19**	**1441**	**5340**

Table S4. Summary of *in silico* metabolic pathway predictions made on genes modulated at a distance by pollination, with comparative enrichment analyses across conditions and clusters. The enzymes codes retrieved from BLAST and Blast2GO analyses were mapped to KEGG metabolic pathways. Columns *Poll.* and *Rest* give the total number of genes regulated in at least one pollination condition, and the number of remaining genes, respectively. Asterisks (*) indicate a significant enrichment for a given prediction in a given sample (Fisher's exact test, $P < 0.05$).

(a) Detailed view of metabolic pathway enrichment in each pollination condition

	6 HAP						24 HAP						48 HAP							
**Metabolic pathway**	**CCP**		**CIP**		**HCP**		**CCP**		**CIP**		**HCP**		**CCP**		**CIP**		**HCP**		**Poll.**	**Rest**
	↑	↓	↑	↓	↑	↓	↑	↓	↑	↓	↑	↓	↑	↓	↑	↓	↑	↓		
**Total ESTs**	**68**	**57**	**57**	**31**	**43**	**21**	**151**	**203**	**52**	**37**	**140**	**145**	**515**	**503**	**65**	**82**	**97**	**69**	**1441**	**5340**
**ESTs involved in metabolic pathways**	12	6	8	4	4	3	10	16	9	1	16	24	51	66	9	12	9	8	180	736
**1. Carbohydrate metabolism**																				
Amino sugar and nucleotide sugar metabolism [00520]	1	1	0	2	0	1	2	1	2	0	2	4	7	4	0	1	2	0	21	64
Ascorbate and aldarate metabolism [00053]	0	1	0	*2	0	1	1	1	0	0	0	0	1	1	0	0	1	0	6	28
Butanoate metabolism [00650]	0	0	0	0	0	0	0	0	0	0	0	2	0	3	0	0	0	1	5	15
C5-Branched dibasic acid metabolism [00660]	0	0	0	0	0	0	0	0	0	0	0	*2	2	0	0	0	0	0	4	8
Citrate cycle (TCA cycle) [00020]	0	0	0	0	0	0	0	0	0	0	0	*3	5	1	0	0	0	1	8	26
Fructose and mannose metabolism [00051]	0	0	0	0	0	0	1	0	0	0	0	0	1	3	0	1	0	0	5	29
Galactose metabolism [00052]	0	0	0	0	0	0	1	0	0	0	0	0	3	1	0	0	0	0	4	*53
Glycolysis / Gluconeogenesis [00010]	1	1	0	2	0	1	1	0	1	0	2	1	3	7	1	1	0	0	13	79
Glyoxylate and dicarboxylate metabolism [00630]	0	0	0	0	0	0	1	0	1	0	2	0	3	5	2	0	0	0	9	35
Inositol phosphate metabolism [00562]	0	0	0	1	0	0	0	0	0	0	0	2	1	1	0	0	0	0	4	24
Pentose and glucuronate interconversions [00040]	0	1	0	*2	0	1	0	1	0	0	1	0	2	2	0	0	1	0	7	32
Pentose phosphate pathway [00030]	1	0	0	0	0	0	0	0	0	0	0	0	2	2	0	1	0	0	4	34
Propanoate metabolism [00640]	0	0	0	0	0	0	0	0	1	0	2	2	2	*8	1	0	0	1	*12	18
Pyruvate metabolism [00620]	0	0	0	0	0	0	0	1	1	0	2	1	4	7	1	0	0	0	12	62
Starch and sucrose metabolism [00500]	1	0	0	0	0	1	0	3	1	0	1	1	2	3	0	1	1	0	10	58
**2. Energy metabolism**																				
Carbon fixation in photosynthetic organisms [00710]	1	0	0	0	0	0	0	0	0	0	0	0	2	4	0	1	0	0	6	33
Carbon fixation pathways in prokaryotes [00720]	0	0	0	0	0	0	0	0	1	0	2	2	5	*7	1	0	0	1	13	27
Methane metabolism [00680]	0	1	0	*2	0	1	1	0	1	0	2	0	3	5	1	1	0	0	10	34
Nitrogen metabolism [00910]	0	0	0	0	0	0	0	0	0	0	0	1	1	1	0	0	0	0	3	13
Oxidative phosphorylation [00190]	0	0	0	1	0	1	1	1	0	1	0	0	0	2	0	0	0	0	4	35
Photosynthesis [00195]	0	0	0	0	0	0	0	0	0	0	0	0	1	1	0	*1	0	0	2	1
Sulfur metabolism [00920]	0	0	1	0	1	0	0	0	0	0	1	0	0	3	0	0	0	0	3	17
**3. Lipid metabolism**																				
alpha-Linolenic acid metabolism [00592]	0	*2	0	*2	0	1	1	2	0	0	0	1	4	4	1	2	0	1	*13	22
Arachidonic acid metabolism [00590]	0	0	0	0	0	0	0	2	0	0	0	2	0	3	0	1	0	0	6	14
Ether lipid metabolism [00565]	0	0	0	0	0	0	0	1	0	0	0	0	0	0	0	1	0	0	1	4
Fatty acid biosynthesis [00061]	0	0	0	0	0	0	0	0	0	0	0	0	1	2	0	0	0	0	3	18
Fatty acid degradation [00071]	0	*2	0	*2	0	1	0	0	0	0	0	0	0	*7	0	0	0	0	10	30
Fatty acid elongation [00062]	0	0	0	0	0	0	0	0	0	0	0	0	0	2	0	0	0	0	2	19
Glycerolipid metabolism [00561]	0	0	0	0	0	0	0	0	0	0	1	2	0	1	0	0	1	0	4	28
Glycerophospholipid metabolism [00564]	0	*2	0	1	0	1	0	2	0	1	2	2	0	2	1	1	2	1	9	30
Linoleic acid metabolism [00591]	0	0	0	0	0	0	0	1	0	0	0	1	0	2	0	*2	0	0	4	9
Sphingolipid metabolism [00600]	0	0	0	0	0	0	0	0	0	0	0	0	2	0	0	0	0	0	2	*47
Steroid biosynthesis [00100]	0	0	0	0	0	0	0	*2	1	0	0	*3	*6	0	0	0	0	0	*8	4
Steroid hormone biosynthesis [00140]	1	0	1	0	0	0	0	0	0	0	0	0	0	1	1	0	0	0	2	17
**4. Nucleotide metabolism**																				
Purine metabolism [00230]	0	0	1	0	1	0	2	2	0	0	3	1	9	7	1	*5	1	1	21	69
Pyrimidine metabolism [00240]	0	0	0	0	0	0	1	0	0	0	0	0	4	1	*2	1	0	0	6	24
**5. Amino acid metabolism**																				
Alanine, aspartate and glutamate metabolism [00250]	0	0	0	0	0	0	0	2	0	0	0	1	0	4	0	0	0	1	6	21
Arginine and proline metabolism [00330]	0	0	0	0	0	0	0	0	1	0	3	0	1	5	0	0	*3	0	9	41
Arginine biosynthesis [00220]	0	0	0	0	0	0	0	0	0	0	0	1	1	2	0	0	0	1	4	16
Cysteine and methionine metabolism [00270]	1	0	1	0	0	0	0	1	0	0	1	1	3	8	0	1	0	0	12	48
Glycine, serine and threonine metabolism [00260]	0	1	0	*2	0	1	1	1	0	0	0	0	3	1	0	0	0	1	7	44
Histidine metabolism [00340]	0	0	0	0	0	0	0	0	0	0	0	0	0	4	0	0	0	0	4	17
Lysine biosynthesis [00300]	0	0	0	0	0	0	0	0	0	0	0	0	0	2	0	0	0	0	2	10
Lysine degradation [00310]	0	0	0	0	0	0	0	0	0	0	0	0	0	3	0	0	0	0	3	32
Phenylalanine metabolism [00360]	0	0	0	0	0	0	0	0	1	0	2	2	2	*11	1	0	0	2	15	38
Phenylalanine, tyrosine and tryptophan biosynthesis [00400]	0	0	0	0	0	0	0	0	0	0	0	0	0	4	0	0	1	0	5	19
Tryptophan metabolism [00380]	0	0	0	0	0	0	0	0	0	0	0	0	1	6	1	0	0	0	8	34
Tyrosine metabolism [00350]	2	1	*2	*2	0	1	0	0	0	0	0	0	1	2	1	0	0	1	8	31
Valine, leucine and isoleucine biosynthesis [00290]	0	0	0	0	0	0	0	0	0	0	0	1	0	0	0	0	0	0	1	15
Valine, leucine and isoleucine degradation [00280]	0	0	0	0	0	0	0	0	0	0	0	0	0	4	0	0	0	0	4	25
**6. Metabolism of other amino acids**																				
beta-Alanine metabolism [00410]	0	0	0	0	0	0	0	0	0	0	0	1	1	3	0	0	0	*2	6	20
Cyanoamino acid metabolism [00460]	0	0	0	0	0	0	1	1	0	0	2	0	3	0	0	1	1	0	6	16
Glutathione metabolism [00480]	0	0	0	0	0	0	2	1	1	0	2	*4	3	8	0	1	1	2	17	43
Selenocompound metabolism [00450]	0	0	1	0	1	0	0	0	0	0	1	0	2	3	0	0	0	0	5	9
Taurine and hypotaurine metabolism [00430]	0	0	0	0	0	0	0	0	0	0	*2	0	1	0	0	0	1	0	2	7

→ *cf.* page suivante

Table S4. (continued)

Metabolic pathway	6 HAP						24 HAP						48 HAP						Poll.	Rest
	CCP		CIP		HCP		CCP		CIP		HCP		CCP		CIP		HCP			
	↑	↓	↑	↓	↑	↓	↑	↓	↑	↓	↑	↓	↑	↓	↑	↓	↑	↓		
**7. Glycan biosynthesis and metabolism**																				
Glycosaminoglycan degradation [00531]	0	0	0	0	0	0	0	0	0	0	0	0	2	0	0	0	0	0	2	27
Glycosphingolipid biosynthesis - ganglio series [00604]	0	0	0	0	0	0	0	0	0	0	0	0	2	0	0	0	0	0	2	26
N-Glycan biosynthesis [00510]	0	0	0	0	0	0	0	0	0	0	0	0	0	1	0	0	0	0	1	9
Other glycan degradation [00511]	0	0	0	0	0	0	1	0	0	0	0	0	3	0	1	1	1	0	4	38
**8. Metabolism of cofactors and vitamins**																				
Folate biosynthesis [00790]	0	0	0	0	0	0	0	0	0	0	0	0	0	2	0	0	0	0	2	15
Nicotinate and nicotinamide metabolism [00760]	0	0	0	0	0	0	1	0	0	0	0	0	2	0	0	0	0	0	2	12
One carbon pool by folate [00670]	0	0	0	0	0	0	1	0	0	0	0	0	3	2	0	0	0	0	5	14
Pantothenate and CoA biosynthesis [00770]	0	0	0	0	0	0	0	0	0	0	0	1	0	1	0	0	0	0	2	19
Porphyrin and chlorophyll metabolism [00860]	0	*2	0	0	0	0	0	2	1	0	0	2	2	2	0	1	0	1	9	21
Retinol metabolism [00830]	0	1	0	*2	0	1	0	0	0	0	0	0	0	1	0	0	0	0	3	17
Riboflavin metabolism [00740]	0	0	0	0	0	0	0	0	0	0	0	0	0	1	0	0	0	0	1	13
Thiamine metabolism [00730]	0	0	0	0	0	0	0	0	0	0	0	0	3	1	0	*2	1	0	4	18
Ubiquinone and other terpenoid-quinone biosynthesis [00130]	*5	0	*4	0	*3	0	0	0	1	0	2	0	0	*6	1	0	0	0	11	22
**9. Metabolism of terpenoids and polyketides**																				
Biosynthesis of vancomycin group antibiotics [01055]	0	0	0	0	0	0	*2	0	*1	0	0	0	*2	0	0	0	0	0	*3	0
Carotenoid biosynthesis [00906]	0	0	0	0	0	0	0	0	0	0	*2	0	0	0	0	0	0	0	2	11
Diterpenoid biosynthesis [00904]	0	0	0	0	0	0	*3	0	0	0	0	0	0	0	*4	0	0	0	*4	1
Geraniol degradation [00281]	0	0	0	0	0	0	0	0	0	0	0	0	0	3	0	0	0	0	3	12
Insect hormone biosynthesis [00981]	0	0	0	0	0	0	0	0	0	0	0	0	0	1	0	0	0	0	1	7
Limonene and pinene degradation [00903]	0	0	0	0	0	0	0	0	0	0	0	0	0	3	0	0	0	0	3	10
Polyketide sugar unit biosynthesis [00523]	0	0	0	0	0	0	*2	0	*1	0	0	0	*2	0	0	0	0	0	*3	0
Terpenoid backbone biosynthesis [00900]	0	0	0	0	0	0	0	1	1	0	0	1	2	3	0	1	0	0	5	9
**10. Biosynthesis of other secondary metabolites**																				
Acarbose and validamycin biosynthesis [00525]	0	0	0	0	0	0	*2	0	*1	0	0	0	*2	0	0	0	0	0	*3	0
Anthocyanin biosynthesis [00942]	0	0	0	0	0	0	0	0	*2	0	0	*2	0	0	0	0	0	*1	2	2
Betalain biosynthesis [00965]	*2	0	*2	0	0	0	0	0	0	0	0	0	0	0	*1	0	0	0	2	1
Caffeine metabolism [00232]	0	0	0	0	0	0	0	0	0	0	0	0	0	1	0	0	0	0	1	10
Flavone and flavonol biosynthesis [00944]	1	0	1	0	0	0	0	0	1	0	0	*2	0	0	1	0	0	1	3	4
Indole alkaloid biosynthesis [00901]	0	0	0	0	0	0	0	0	0	0	0	0	1	0	0	1	0	0	1	6
Isoquinoline alkaloid biosynthesis [00950]	1	0	1	0	0	0	0	0	0	0	0	1	1	2	1	0	0	*2	7	11
Monobactam biosynthesis [00261]	0	0	*1	0	*1	0	0	0	0	0	1	0	0	*2	0	0	0	0	2	2
Novobiocin biosynthesis [00401]	0	0	0	0	0	0	0	0	0	0	0	0	0	2	0	0	0	0	2	5
Penicillin and cephalosporin biosynthesis [00311]	0	0	0	0	0	0	0	0	0	0	0	0	1	1	0	0	0	0	2	14
Phenylpropanoid biosynthesis [00940]	1	1	0	0	0	0	1	2	1	0	2	2	4	8	1	0	0	2	16	46
Streptomycin biosynthesis [00521]	0	0	0	0	0	0	*2	0	1	0	0	0	2	0	0	0	0	0	3	9
Tropane, piperidine and pyridine alkaloid biosynthesis [00960]	0	0	0	0	0	0	0	0	1	0	0	0	1	2	0	1	0	1	5	13
**11. Xenobiotics biodegradation and metabolism**																				
Aminobenzoate degradation [00627]	*5	0	*4	0	*3	0	0	0	1	0	0	0	2	4	0	0	0	0	12	21
Benzoate degradation [00362]	1	0	0	0	0	0	0	0	0	0	0	0	0	2	0	0	0	0	3	12
Caprolactam degradation [00930]	0	0	0	0	0	0	0	0	0	0	0	0	0	2	0	0	0	0	2	10
Chloroalkane and chloroalkene degradation [00625]	0	1	0	*2	0	1	0	1	0	0	0	1	0	1	0	0	0	0	4	13
Chlorocyclohexane and chlorobenzene degradation [00361]	*6	0	*4	0	*3	0	0	0	0	0	0	0	0	0	0	0	0	0	*6	5
Drug metabolism - cytochrome P450 [00982]	0	1	0	*2	0	1	1	1	0	0	0	3	1	3	0	0	0	2	8	32
Drug metabolism - other enzymes [00983]	0	0	0	0	0	0	2	1	0	0	1	3	6	4	2	1	0	2	14	41
Fluorobenzoate degradation [00364]	*1	0	0	0	0	0	0	0	0	0	0	0	0	0	0	0	0	0	1	2
Metabolism of xenobiotics by cytochrome P450 [00980]	0	1	0	*2	0	1	1	1	0	0	0	3	1	3	0	0	0	2	8	34
Naphthalene degradation [00626]	0	1	0	*2	0	*1	0	0	0	0	0	0	0	0	0	0	0	0	2	5
Styrene degradation [00643]	0	0	0	0	0	0	0	0	0	0	0	0	1	1	0	0	0	0	2	9
Toluene degradation [00623]	1	0	0	0	0	0	0	0	0	0	0	0	0	0	0	0	0	0	1	7

Table S4. (continued)

(b) Detailed view of metabolic pathway enrichment in each cluster

Prediction	Clusters																									Poll.	Rest
	1	2	3	4	5	6	7	8	9	10	11	12	13	14	15	16	17	18	19	20	21	22	23	24	25		
**Total ESTs**	**34**	**35**	**15**	**110**	**65**	**80**	**32**	**163**	**83**	**36**	**33**	**33**	**52**	**260**	**60**	**72**	**10**	**48**	**5**	**97**	**18**	**43**	**25**	**13**	**19**	**1441**	**5340**
**ESTs involved in metabolic pathways**	5	7	0	11	10	5	3	28	10	8	2	7	6	25	6	0	3	5	1	***24**	1	0	7	3	3	180	736
**1. Carbohydrate metabolism**																											
Amino sugar and nucleotide sugar metabolism [00520]	2	2	0	0	0	0	0	2	2	0	0	0	0	4	2	0	0	***3**	0	2	0	0	0	***2**	0	21	64
Ascorbate and aldarate metabolism [00053]	1	0	0	0	0	0	0	1	0	0	0	0	1	1	0	0	0	0	0	0	0	0	0	***2**	0	6	28
Butanoate metabolism [00650]	0	0	0	0	1	0	0	2	0	0	0	0	0	0	0	0	0	1	0	1	0	0	0	0	0	5	15
C5-Branched dibasic acid metabolism [00660]	0	0	0	0	0	0	0	0	0	0	0	0	1	0	1	0	0	0	0	***2**	0	0	0	0	0	4	8
Citrate cycle (TCA cycle) [00020]	0	0	0	0	1	0	0	0	0	1	0	0	***2**	1	1	0	0	0	0	2	0	0	0	0	0	8	26
Fructose and mannose metabolism [00051]	0	0	0	1	0	0	0	0	2	0	0	0	0	1	0	0	0	0	0	0	0	1	0	0	0	5	29
Galactose metabolism [00052]	0	0	0	0	0	0	0	0	0	1	0	0	1	2	0	0	0	0	0	0	0	0	0	0	0	4	***53**
Glycolysis / Gluconeogenesis [00010]	0	0	0	1	0	0	0	5	0	2	0	0	2	0	1	0	0	0	0	0	0	0	0	***2**	0	13	79
Glyoxylate and dicarboxylate metabolism [00630]	0	0	0	0	0	0	0	***4**	0	0	0	0	0	3	0	0	0	0	0	0	0	0	1	0	1	9	35
Inositol phosphate metabolism [00562]	0	0	0	0	0	0	0	1	0	0	0	0	0	0	1	0	0	0	***1**	1	0	0	0	0	0	4	24
Pentose and glucuronate interconversions [00040]	0	1	0	0	1	0	1	0	0	0	0	0	0	1	1	0	0	0	0	0	0	0	0	***2**	0	7	32
Pentose phosphate pathway [00030]	0	0	0	1	0	0	0	0	0	0	1	0	1	1	0	0	0	0	0	0	0	0	0	0	0	4	34
Propanoate metabolism [00640]	0	0	0	0	1	0	0	***7**	0	0	0	0	1	0	1	0	0	1	0	1	0	0	0	0	0	***12**	18
Pyruvate metabolism [00620]	0	0	0	0	1	0	0	***6**	0	1	0	0	1	2	1	0	0	0	0	0	0	0	0	0	0	12	62
Starch and sucrose metabolism [00500]	***3**	0	0	1	1	1	0	1	0	0	0	0	0	2	0	0	0	0	0	1	0	0	0	0	0	10	58
**2. Energy metabolism**																											
Carbon fixation in photosynthetic organisms [00710]	0	0	0	1	0	0	0	1	1	0	0	0	1	1	0	0	0	0	0	0	0	0	0	0	1	6	33
Carbon fixation pathways in prokaryotes [00720]	0	0	0	0	1	0	0	***6**	0	0	0	0	***2**	1	1	0	0	0	0	2	0	0	0	0	0	13	27
Methane metabolism [00680]	0	0	0	1	0	0	0	***4**	0	0	0	0	0	3	0	0	0	0	0	0	0	0	0	***2**	0	10	34
Nitrogen metabolism [00910]	0	0	0	0	1	0	0	0	0	0	0	0	0	1	0	0	0	0	0	1	0	0	0	0	0	3	13
Oxidative phosphorylation [00190]	0	0	1	0	1	0	0	0	0	***2**	0	0	0	0	0	0	0	0	0	0	0	0	0	0	0	4	35
Photosynthesis [00195]	0	0	0	0	0	***1**	0	0	0	0	0	0	0	1	0	0	0	0	0	0	0	0	0	0	0	2	1
Sulfur metabolism [00920]	0	0	0	0	0	0	0	2	0	0	0	0	0	0	0	0	0	0	0	0	0	0	1	0	0	3	17
**3. Lipid metabolism**																											
alpha-Linolenic acid metabolism [00592]	0	0	0	1	0	0	0	3	0	1	0	0	0	2	0	0	0	1	0	2	0	0	1	***2**	0	***13**	22
Arachidonic acid metabolism [00590]	0	0	0	1	1	0	0	1	0	0	1	0	0	0	0	0	***2**	0	0	0	0	0	0	0	0	6	14
Ether lipid metabolism [00565]	0	0	0	1	0	0	0	0	0	0	0	0	0	0	0	0	0	0	0	0	0	0	0	0	0	1	4
Fatty acid biosynthesis [00061]	0	0	0	0	1	1	0	0	0	0	0	0	0	1	0	0	0	0	0	0	0	0	0	0	0	3	18
Fatty acid degradation [00071]	0	0	0	0	2	1	0	3	0	0	1	0	0	0	0	0	0	0	0	0	0	0	0	***2**	1	10	30
Fatty acid elongation [00062]	0	0	0	0	0	0	0	2	0	0	0	0	0	0	0	0	0	0	0	0	0	0	0	0	0	2	19
Glycerolipid metabolism [00561]	0	0	0	1	0	0	0	1	0	0	0	0	0	0	0	0	0	0	0	2	0	0	0	0	0	4	28
Glycerophospholipid metabolism [00564]	0	1	0	2	0	0	0	2	0	0	0	0	0	0	0	0	0	0	0	2	1	0	1	0	0	9	30
Linoleic acid metabolism [00591]	0	0	0	1	1	0	0	0	0	1	0	0	0	0	0	0	0	1	0	0	0	0	0	0	0	4	9
Sphingolipid metabolism [00600]	0	0	0	0	0	0	0	0	0	0	0	0	0	2	0	0	0	0	0	0	0	0	0	0	0	2	***47**
Steroid biosynthesis [00100]	0	0	0	***2**	0	0	0	0	0	0	0	0	0	0	0	0	0	0	0	***6**	0	0	0	0	0	***8**	4
Steroid hormone biosynthesis [00140]	0	0	0	0	1	0	0	0	0	0	0	1	0	0	0	0	0	0	0	0	0	0	0	0	0	2	17
**4. Nucleotide metabolism**																											
Purine metabolism [00230]	0	0	0	4	1	0	0	3	2	0	0	0	0	6	1	1	0	1	0	0	1	1	0	0	0	21	69
Pyrimidine metabolism [00240]	0	0	0	0	0	0	0	1	0	0	0	0	0	3	0	0	0	0	0	1	0	0	1	0	0	6	24
**5. Amino acid metabolism**																											
Alanine, aspartate and glutamate metabolism [00250]	0	0	0	0	0	0	0	2	***2**	0	0	0	0	0	0	0	***1**	1	0	0	0	0	0	0	0	6	21
Arginine and proline metabolism [00330]	1	0	0	2	0	1	0	2	1	1	0	0	0	1	0	0	0	0	0	0	0	0	0	0	0	9	41
Arginine biosynthesis [00220]	0	0	0	0	0	0	0	1	1	0	0	0	0	1	0	0	0	1	0	0	0	0	0	0	0	4	16
Cysteine and methionine metabolism [00270]	0	1	0	0	0	0	0	3	2	1	1	0	0	2	0	0	0	0	0	1	0	0	1	0	0	12	48
Glycine, serine and threonine metabolism [00260]	0	0	0	0	0	0	0	0	1	0	0	0	0	2	0	0	0	0	0	2	0	0	0	***2**	0	7	44
Histidine metabolism [00340]	0	0	0	0	0	0	0	2	***2**	0	0	0	0	0	0	0	0	0	0	0	0	0	0	0	0	4	17
Lysine biosynthesis [00300]	0	0	0	0	0	0	0	1	1	0	0	0	0	0	0	0	0	0	0	0	0	0	0	0	0	2	10
Lysine degradation [00310]	0	0	0	0	0	0	0	3	0	0	0	0	0	0	0	0	0	0	0	0	0	0	0	0	0	3	32
Phenylalanine metabolism [00360]	1	0	0	0	0	0	0	***7**	2	1	0	0	0	1	0	0	0	1	0	2	0	0	0	0	0	15	38
Phenylalanine, tyrosine and tryptophan biosynth. [00400]	0	0	0	1	0	0	***2**	1	1	0	0	0	0	0	0	0	0	0	0	0	0	0	0	0	0	5	19
Tryptophan metabolism [00380]	1	0	0	0	1	0	0	3	1	0	0	0	0	1	0	0	0	0	0	0	0	0	1	0	0	8	34
Tyrosine metabolism [00350]	0	0	0	0	0	0	0	1	1	0	0	***2**	0	0	0	0	0	0	0	2	0	0	0	***2**	0	8	31
Valine, leucine and isoleucine biosynthesis [00290]	0	0	0	0	0	0	0	0	0	0	0	0	0	0	0	0	0	0	0	1	0	0	0	0	0	1	15
Valine, leucine and isoleucine degradation [00280]	0	0	0	0	1	0	0	***3**	0	0	0	0	0	0	0	0	0	0	0	0	0	0	0	0	0	4	25
**6. Metabolism of other amino acids**																											
beta-Alanine metabolism [00410]	0	0	0	0	0	0	0	***3**	0	0	0	0	0	0	0	0	0	1	0	2	0	0	0	0	0	6	20
Cyanoamino acid metabolism [00460]	***2**	***2**	0	0	0	0	0	0	0	0	0	0	0	2	0	0	0	0	0	0	0	0	0	0	0	6	16
Glutathione metabolism [00480]	0	***2**	0	0	2	0	0	4	0	1	***2**	0	1	1	0	0	1	0	0	2	0	1	0	0	0	17	43
Selenocompound metabolism [00450]	0	0	0	0	0	0	0	***2**	0	0	0	0	0	2	0	0	0	0	0	0	0	0	1	0	0	5	9
Taurine and hypotaurine metabolism [00430]	0	***2**	0	0	0	0	0	0	0	0	0	0	0	0	0	0	0	0	0	0	0	0	0	0	0	2	7

Table S4. (continued)

Prediction	Clusters																									Poll.	Rest
	1	2	3	4	5	6	7	8	9	10	11	12	13	14	15	16	17	18	19	20	21	22	23	24	25		
**7. Glycan biosynthesis and metabolism**																											
Glycosaminoglycan degradation [00531]	0	0	0	0	0	0	0	0	0	0	0	0	0	2	0	0	0	0	0	0	0	0	0	0	0	2	27
Glycosphingolipid biosynthesis - ganglio series [00604]	0	0	0	0	0	0	0	0	0	0	0	0	0	2	0	0	0	0	0	0	0	0	0	0	0	2	26
N-Glycan biosynthesis [00510]	0	0	0	0	0	1	0	0	0	0	0	0	0	0	0	0	0	0	0	0	0	0	0	0	0	1	9
Other glycan degradation [00511]	1	0	0	0	0	0	0	0	0	0	0	0	0	2	0	0	0	0	0	0	1	0	0	0	0	4	38
**8. Metabolism of cofactors and vitamins**																											
Folate biosynthesis [00790]	0	0	0	0	0	0	0	2	0	0	0	0	0	0	0	0	0	0	0	0	0	0	0	0	0	2	15
Nicotinate and nicotinamide metabolism [00760]	0	0	0	0	0	0	0	0	0	0	0	0	1	1	0	0	0	0	0	0	0	0	0	0	0	2	12
One carbon pool by folate [00670]	0	0	0	0	0	0	0	2	0	0	0	0	0	*3	0	0	0	0	0	0	0	0	0	0	0	5	14
Pantothenate and CoA biosynthesis [00770]	0	0	0	0	0	0	0	1	0	0	0	0	0	0	0	0	0	0	0	1	0	0	0	0	0	2	19
Porphyrin and chlorophyll metabolism [00860]	1	0	0	1	1	0	0	1	0	0	0	0	1	0	0	0	0	0	0	2	0	0	0	0	*2	9	21
Retinol metabolism [00830]	0	0	0	0	1	0	0	0	0	0	0	0	0	0	0	0	0	0	0	0	0	0	0	*2	0	3	17
Riboflavin metabolism [00740]	0	0	0	0	0	0	0	1	0	0	0	0	0	0	0	0	0	0	0	0	0	0	0	0	0	1	13
Thiamine metabolism [00730]	0	0	0	0	0	0	0	1	0	0	0	0	0	3	0	0	0	0	0	0	0	0	0	0	0	4	18
Ubiquinone and other terpenoid-quinone biosynth. [00130]	0	0	0	0	0	0	0	*5	1	0	0	*4	0	0	0	0	0	0	0	1	0	0	0	0	0	11	22
**9. Metabolism of terpenoids and polyketides**																											
Biosynthesis of vancomycin group antibiotics [01055]	0	0	0	0	0	0	0	0	0	0	0	0	0	1	*1	0	0	0	0	*1	0	0	0	0	0	*3	0
Carotenoid biosynthesis [00906]	0	*2	0	0	0	0	0	0	0	0	0	0	0	0	0	0	0	0	0	0	0	0	0	0	0	2	11
Diterpenoid biosynthesis [00904]	0	0	0	0	0	0	0	0	0	0	0	0	0	0	0	0	0	0	0	0	0	0	*4	0	0	*4	1
Geraniol degradation [00281]	0	0	0	0	1	0	0	*2	0	0	0	0	0	0	0	0	0	0	0	0	0	0	0	0	0	3	12
Insect hormone biosynthesis [00981]	0	0	0	0	0	0	0	1	0	0	0	0	0	0	0	0	0	0	0	0	0	0	0	0	0	1	7
Limonene and pinene degradation [00903]	0	0	0	0	0	0	0	*3	0	0	0	0	0	0	0	0	0	0	0	0	0	0	0	0	0	3	10
Polyketide sugar unit biosynthesis [00523]	0	0	0	0	0	0	0	0	0	0	0	0	0	1	*1	0	0	0	0	*1	0	0	0	0	0	*3	0
Terpenoid backbone biosynthesis [00900]	0	0	0	0	0	0	0	0	*3	0	0	0	0	0	0	0	0	0	0	*2	0	0	0	0	0	5	9
**10. Biosynthesis of other secondary metabolites**																											
Acarbose and validamycin biosynthesis [00525]	0	0	0	0	0	0	0	0	0	0	0	0	0	1	*1	0	0	0	0	*1	0	0	0	0	0	*3	0
Anthocyanin biosynthesis [00942]	0	0	0	0	0	0	0	0	0	0	0	0	0	0	0	0	0	0	0	*2	0	0	0	0	0	2	2
Betalain biosynthesis [00965]	0	0	0	0	0	0	0	0	0	0	0	*2	0	0	0	0	0	0	0	0	0	0	0	0	0	2	1
Caffeine metabolism [00232]	0	0	0	0	1	0	0	0	0	0	0	0	0	0	0	0	0	0	0	0	0	0	0	0	0	1	10
Flavone and flavonol biosynthesis [00944]	0	0	0	0	0	0	0	0	0	0	0	*1	0	0	0	0	0	0	0	*2	0	0	0	0	0	3	4
Indole alkaloid biosynthesis [00901]	0	0	0	0	0	0	0	0	0	0	0	0	0	0	0	1	0	0	0	0	0	0	0	0	0	1	6
Isoquinoline alkaloid biosynthesis [00950]	0	0	0	0	0	0	0	1	1	0	0	1	0	0	0	0	0	0	0	*3	0	0	1	0	0	7	11
Monobactam biosynthesis [00261]	0	0	0	0	0	0	0	*2	0	0	0	0	0	0	0	0	0	0	0	0	0	0	0	0	0	2	2
Novobiocin biosynthesis [00401]	0	0	0	0	0	0	0	1	1	0	0	0	0	0	0	0	0	0	0	0	0	0	0	0	0	2	5
Penicillin and cephalosporin biosynthesis [00311]	0	0	0	0	0	0	0	0	1	0	0	0	0	1	0	0	0	0	0	0	0	0	0	0	0	2	14
Phenylpropanoid biosynthesis [00940]	1	0	0	0	2	0	0	*6	0	1	0	0	0	3	1	0	0	0	0	1	0	0	0	1	0	16	46
Streptomycin biosynthesis [00521]	0	0	0	0	0	0	0	0	0	0	0	0	0	1	1	0	0	0	0	1	0	0	0	0	0	3	9
Tropane, piperidine and pyridine alkaloid biosynth. [00960]	0	0	0	0	0	0	0	1	1	0	0	0	0	0	0	0	*1	0	0	*2	0	0	0	0	0	5	13
**11. Xenobiotics biodegradation and metabolism**																											
Aminobenzoate degradation [00627]	1	0	0	0	1	0	0	2	0	1	0	*4	0	1	1	0	0	0	0	1	0	0	0	0	0	12	21
Benzoate degradation [00362]	0	0	0	0	0	0	0	*2	0	0	0	1	0	0	0	0	0	0	0	0	0	0	0	0	0	3	12
Caprolactam degradation [00930]	0	0	0	0	0	0	0	*2	0	0	0	0	0	0	0	0	0	0	0	0	0	0	0	0	0	2	10
Chloroalkane and chloroalkene degradation [00625]	0	0	0	0	0	0	0	1	0	0	0	0	0	0	0	0	*1	0	0	0	0	0	0	*2	0	4	13
Chlorocyclohexane and chlorobenzene degradation [00361]	0	0	0	0	0	0	0	0	0	0	0	*5	0	0	0	0	0	0	0	1	0	0	0	0	0	*6	5
Drug metabolism - cytochrome P450 [00982]	0	0	0	0	2	0	0	1	0	0	0	0	0	0	0	0	0	0	0	2	0	1	0	*2	0	8	32
Drug metabolism - other enzymes [00983]	0	1	0	0	1	0	0	1	1	0	0	0	0	3	0	0	0	0	0	*4	0	1	*2	0	0	14	41
Fluorobenzoate degradation [00364]	0	0	0	0	0	0	0	0	0	0	0	*1	0	0	0	0	0	0	0	0	0	0	0	0	0	1	2
Metabolism of xenobiotics by cytochrome P450 [00980]	0	0	0	0	2	0	0	1	0	0	0	0	0	0	0	0	0	0	0	2	0	1	0	*2	0	8	34
Naphthalene degradation [00626]	0	0	0	0	0	0	0	0	0	0	0	0	0	0	0	0	0	0	0	0	0	0	0	*2	0	2	5
Styrene degradation [00643]	1	0	0	0	0	0	0	0	0	0	0	0	0	1	0	0	0	0	0	0	0	0	0	0	0	2	9
Toluene degradation [00623]	0	0	0	0	0	0	0	0	0	0	0	*1	0	0	0	0	0	0	0	0	0	0	0	0	0	1	7
